# The role of natural products in the intestinal barrier: a bibliometric analysis from 2004 to 2025

**DOI:** 10.3389/fimmu.2026.1936472

**Published:** 2026-09-01

**Authors:** Hanzhang Hong, Yuxin Hu, ChongZhi Wang, Xiaoyun Zhu

**Affiliations:** 1 Dongzhimen Hospital, Beijing University of Chinese Medicine, Beijing, China; 2 Guang’anmen Hospital, China Academy of Chinese Medical Sciences, Beijing, China

**Keywords:** bibliometric analysis, gut microbiota, inflammation, intestinal barrier, natural products

## Abstract

**Background:**

There is an increasing number of studies on the protective effects of natural products on the intestinal barrier through multiple targets. However, the landscape of knowledge structure and emerging frontiers remains incompletely characterized. We conducted a bibliometric analysis and mapped the global landscape of natural products in intestinal barrier protection research.

**Methods:**

Using a predefined topic search strategy, we retrieved publications from the Web of Science Core Collection and PubMed from January 1, 2004 to November 6, 2025. We analyzed the complete records and citing literature using CiteSpace, VOSviewer, R (Biblioshiny), and SCImago Graphica to quantify the annual output and collaboration of countries/institutions/authors, and analyzed the influence of journals and publications through citation and co-citation analysis. We also detected the evolution of themes in the field through isomorphic keyword co-occurrence, clustering, and burst detection.

**Results:**

We included 1,679 records. 63 countries contributed to the development of this field, with China leading in productivity/paper output and influence (682 publications; 15,987 citations), followed by the United States and the United Kingdom. High-impact publications include Food & Function and Nutrients. Keyword analysis emphasizes that the gut microbiota, inflammation, and oxidative stress are the central pillars, and NF-κB signaling is the key mechanism hub. Polyphenols are the most studied category, and resveratrol is a representative cross-mechanism compound.

**Conclusion:**

The research scale in this field is expanding rapidly and is centered on the gut microbiota, linking immune inflammation and oxidative stress pathways with tight junction integrity and mucosal repair. Future work should strengthen cross-national cooperation and prioritize rigorous translational and clinical validation.

## 1 Introduction

The intestinal barrier constitutes the interface between the mucosal immune system of the lamina propria and the external environment of the intestinal lumen, representing the site exposed to the highest antigenic load derived from luminal microorganisms and dietary antigens (1). Its primary function is to prevent the uncontrolled translocation of microorganisms and antigens across the intestinal wall while permitting bidirectional regulation between luminal factors and the host immune system (2–4). The intestinal barrier is composed of multiple coordinated layers. The epithelial monolayer and its apical junctional complex, particularly tight junctions (TJs), determine the permeability threshold of the paracellular pathway. The mucus layer secreted by goblet cells, with the MUC2 polymeric network serving as its structural scaffold, spatially separates bacteria from the epithelium and limits bacterial adhesion. Antimicrobial peptides produced by Paneth cells and epithelial cells, together with secretory immunoglobulin A (sIgA), constitute the chemical and immunological exclusion barrier. Meanwhile, the immune cell network within the lamina propria maintains a dynamic balance between tolerance to commensal microbiota and rapid responses to invading pathogens (5–9). Disruption of any of these components—including epithelial junctions, the mucus layer, or the antimicrobial/immune exclusion system—can result in a leaky gut phenotype, facilitating the translocation of luminal pathogen-associated molecular patterns (PAMPs) and damage-associated molecular patterns (DAMPs) into the mucosa and even the systemic circulation, thereby increasing the aberrant or persistent activation of host innate immune receptors (10). Accumulating evidence indicates that impairment of the intestinal barrier is a key contributor to inflammatory bowel disease, obesity, metabolic disorders, and other pathological conditions (11).

Among the mechanisms regulating intestinal barrier function, natural products have attracted considerable attention because they frequently exert pleiotropic effects by simultaneously stabilizing epithelial junctions and modulating inflammatory signaling and cellular stress responses, making them valuable candidates for investigating the combined effects of anti-inflammation and tissue repair. For example, urolithin A (Uro A) enhances intestinal barrier function by promoting mucin synthesis while simultaneously alleviating inflammation. Mulberry bark-derived exosome-like nanoparticles (MBELNs) enhance mucus barrier function and ameliorate intestinal inflammation in mice (12). Poly(lactic-co-glycolic acid)/poly(lactic acid)-polyethylene glycol-folic acid (PLGA/PLA-PEG-FA) nanoparticles loaded with the ginger-derived bioactive compound 6-shogaol accelerate colonic wound healing in experimental colitis by downregulating pro-inflammatory cytokines, including TNF-α and IL-6, while activating the Nrf2/HO-1 stress-responsive antioxidant pathway (13). Curcumin improves the structure and function of TJs and attenuates lipopolysaccharide (LPS)-induced inflammatory responses (14). Berberine not only suppresses macrophage and granulocyte activation but also promotes epithelial regeneration by activating Lgr5^+^ intestinal stem cells (ISCs) (15). In addition, (−)-epigallocatechin-3-gallate (EGCG) stabilizes the intestinal barrier and protects against barrier dysfunction induced by pro-inflammatory cytokines (16).

In recent years, research on the role of natural products in intestinal barrier function has continued to expand with respect to both experimental models and mechanistic insights. However, a comprehensive assessment of the current research landscape and future directions in this field, as well as an objective characterization of its major research priorities, remains lacking. Bibliometric analysis is an effective approach for evaluating the overall development trends within a research field. It not only enables researchers and clinicians to identify the leading countries, institutions, authors, and influential publications but also facilitates the identification of thematic evolution, emerging research hotspots, and existing knowledge gaps (17). This approach has been widely applied across diverse disciplines, including medical research (18, 19). To our knowledge, bibliometric studies have investigated the roles of natural products in areas such as therapeutics for cognitive impairment (20), liver diseases (21), and diabetic kidney disease (22), whereas no bibliometric analysis has specifically focused on natural products in relation to intestinal barrier function. Therefore, the present study aimed to employ bibliometric analysis to systematically characterize the current research status and developmental trends of natural products in the field of intestinal barrier function, thereby providing a scientific reference for future drug development and the treatment of intestinal barrier-related diseases.

## 2 Methods

### Data source and search strategy

In this study, WoSCC was selected as the main database because it has high-quality indexes, standardized literature records, and is widely used in bibliometric analysis. Therefore, Web of Science Core Collection (WoSCC) is used as our research database. PubMed was searched to supplement the clinical research landscape, ensuring data comprehensiveness and methodological rigor.This study comprehensively collected all relevant publications published from January 1, 2004 to November 6, 2025. The search formula can be viewed in the supplements.

We retrieved a total of 1,973 records and exported the complete records and citation references in plain text file format, and finally 1,679 records were included. Among them, 1,475 articles were included in WOS, 498 articles in PubMed, and 294 irrelevant and duplicate articles. Among them, there were 231 duplicate articles and 63 irrelevant articles. We believe that this analysis of this time period can reflect the research trends in this field, aiming to enable other scholars to present the role of natural substances in the intestinal barrier from a more rigorous and comprehensive perspective.

### Data collection and quality assessment

We explain our analysis results using the H-index and the IF index. The H-index is used to estimate the significance and wide influence of a scientist’s cumulative research contributions, and has become a well-known indicator for evaluating the research influence of academic scholars (21). The H-index was invented by Jorge Eduardo Hirsch, an Argentine-American physics professor from the University of California, San Diego, in 2005. If an author’s Np papers have H papers that are cited at least H times, and the other (Np - H) papers are each cited ≤ H times, then the scientist’s index is H (23). The IF is often used as a measure of journal quality, and research published in journals with high IF indices often represents higher academic value (24).

### Bibliometric analysis and visualization

In this study, we employed Citespace (6.1.R6), VOSviewer (1.6.20.0), R Studio (4.3.3), and SCImago Graphica Beta (1.0.45) for data visualization analysis. When running Citespace, we chose the R package ggplot2 (3.4.2) to analyze the burst of keywords and clustering situations. Using VOSviewer, we analyzed the distribution of publishing institutions and authors, as well as the frequency of collaboration, and the co-citation situation of keywords and journals. At the same time, we used the data exported from VOSviewer to conduct geographical visualization analysis in SCImago, to analyze the cooperation among countries. When running R, we used Biblioshiny to visualize the changes in publications over time and the distribution of publications in different journals.

VOSviewer was developed by Van Eck and Waltman from the Science and Technology Research Center of Leiden University in the Netherlands in 2009. It is a free software based on Java, with strong graphical capabilities, suitable for handling large-scale data, and focused on the visualization of bibliometric networks. It can assign a group of closely related nodes to multiple clusters, where the same color indicates a higher correlation of the nodes (25). Additionally, VOSviewer supports overlay visualization maps, where the color and distance of nodes represent the distribution pattern of nodes in a two-dimensional space, namely time and association (26). The SCImago Graphica software does not have functions for analyzing network nodes, calculating paths, and clustering, but by combining it with visualization software for text mining in science, it can conduct cooperative network analysis (countries, institutions, authors), keyword co-occurrence analysis, and geographical visualization analysis, etc.

## 3 Result

### 3.1An Overview of publications

The number of publications over a certain period can reflect the research speed and trend in a particular field (27). From January 1, 2004 to November 6, 2025, a total of 1,679 publications related to the role of natural products in the intestinal barrier function were identified.

**Figure 1:**
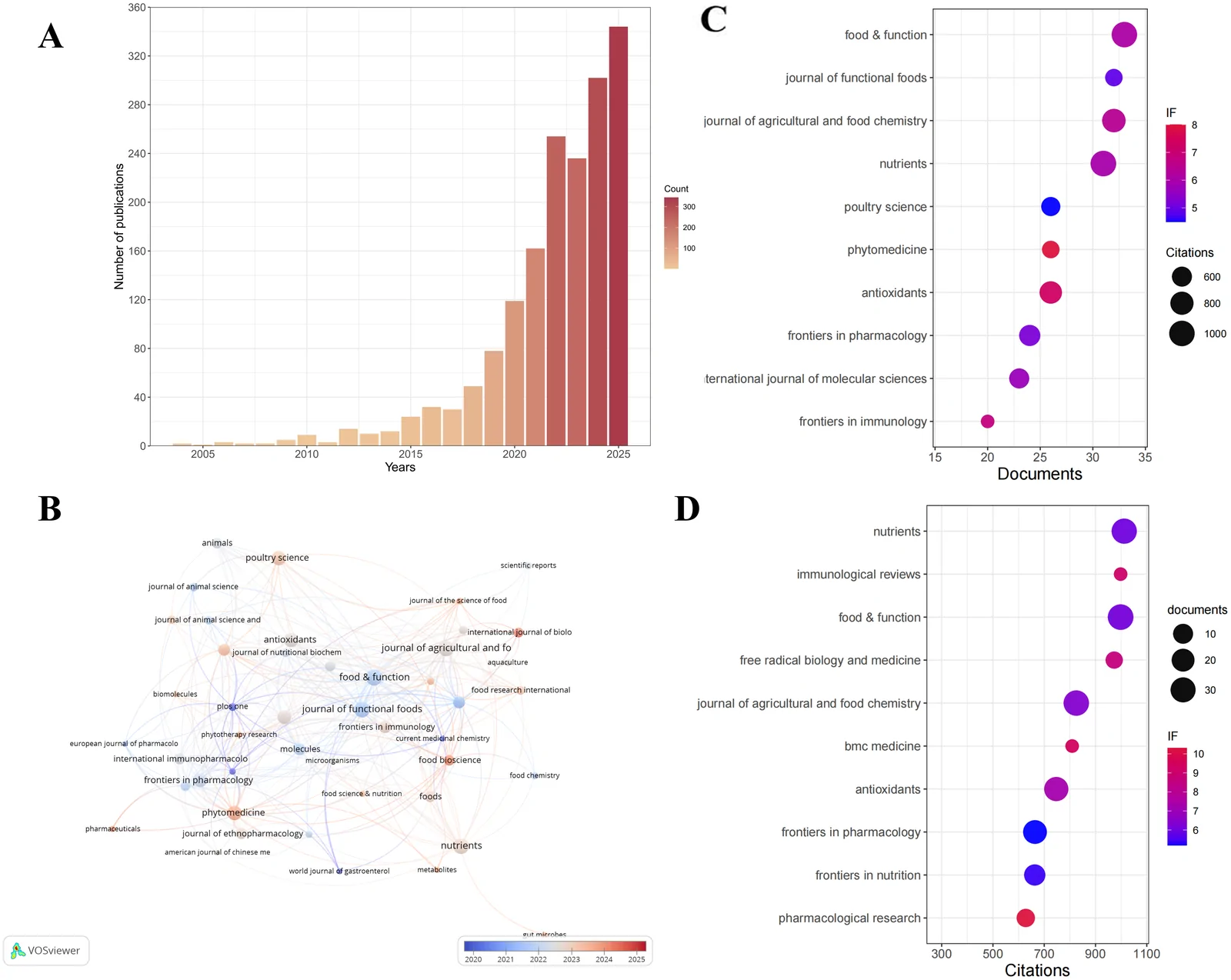
Temporal distribution map of the literature. **(A)** Global trends of annual publications and H-index related to the role of natural products in intestinal barrier function from 2004 to 2025. **(B)** Co-citation analysis of journals where publications related to the role of natural products in intestinal barrier function are located. **(C)** Top 10 journals by publication ranking and their IF indices from 2004 to 2025. **(D)** Top 10 journals by citation count and their IF indices from 2004 to 2025. H-index, The Hirsch index, also known as the H-index, is used to estimate the importance and broad impact of scientists’ cumulative research contributions. IF, impact factors; TC, Total citation.

The number of publications in this field has basically continued to increase from 2004 to 2025, and it has increased rapidly since 2020, indicating that the current research enthusiasm is continuously high. The Mean TC per year has increased after 2010, indicating that the research in this field has begun to receive more attention. This reflects that the research on natural products and intestinal barrier function has become a leading hotspot in the fields of life science and medicine (**Figures 1A, B**).

In terms of citation frequency, Nutrients has the highest number of citations (1,012 times, IF = 6.0), and this journal has the highest impact level among all, with many reviews and wide dissemination (**Figure 1C**). The next are Food & Function (998 times, IF = 6.1) and Immunological Reviews (998 times, IF = 9.1). Food & Function is also the most actively published journal, mainly focusing on research on food-derived components and having a stronger professionalism (**Figure 1D**).

### 3.2 National contributions and international collaboration

**Figure 2:**
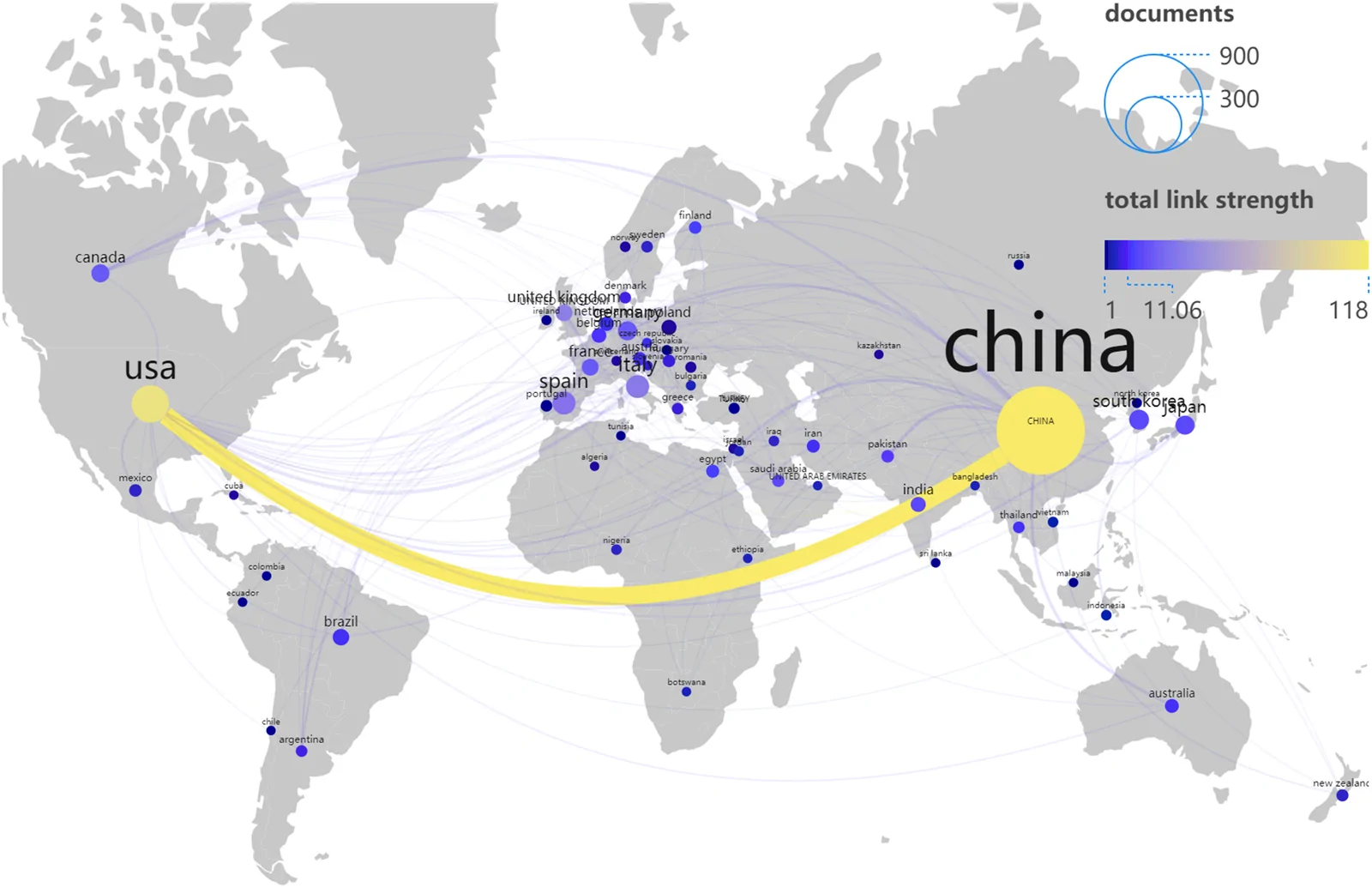
National contributions and international collaboration. World country map. Each node represents a country. The size of the node is positively correlated with the number of published articles. The color of the node is positively correlated with the intensity of cooperation. The intensity of cooperation is quantified by the total link strength calculated by VOSviewer.

A total of 63 countries have published articles, contributing to the research on the role of natural products in the intestinal barrier function. **Figure 2** shows that cooperative connections have been established among many countries (**Table 1**). Among them, China has the most extensive cooperation scope, followed by the USA and the United Kingdom. China has the highest number of publications and citations, indicating that China has made outstanding contributions to the development of this field. **Table 1** shows the top 10 countries in terms of the number of cooperative publications and their publication volumes and citation counts.

**Table 1:** Co-country analysis.

No.	Country	Total intensity of cooperation	Documents	Citations
1	china	118	682	15987
2	usa	108	116	6077
3	united kingdom	36	18	1530
4	italy	34	40	2073
5	spain	31	40	2534
6	canada	21	22	878
7	france	21	18	860
8	germany	21	25	1647
9	japan	17	25	966
10	south korea	16	28	431

### 3.3 Institutional research output

**Figure 3:**
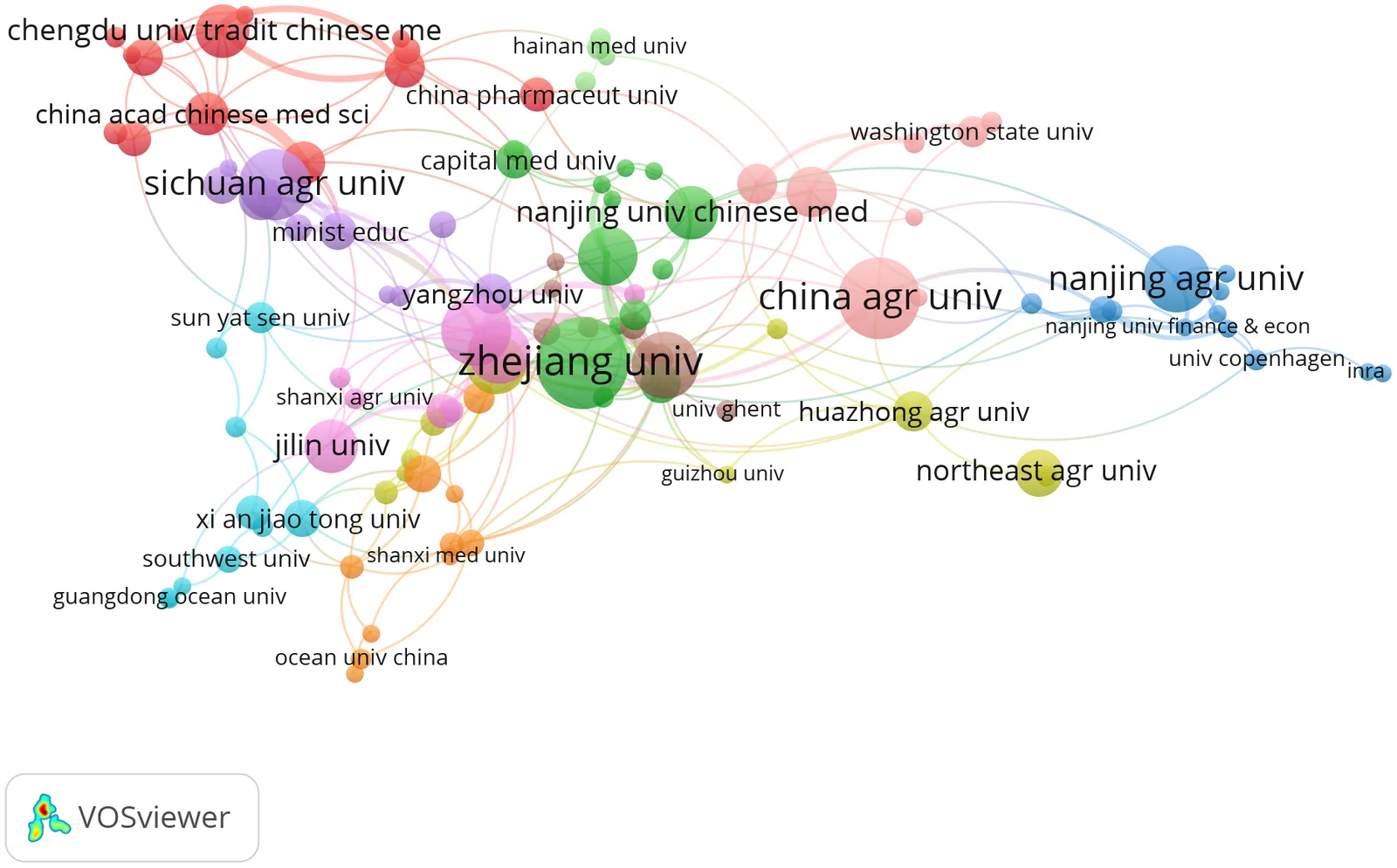
Visualization of organizations cooperation. Each node represents an organization. The node size is positively correlated with the number of published articles, the node color is related to time, and the thickness of the lines between nodes is positively correlated with the frequency of cooperation.

A total of 1,296 institutions from 63 countries published their research (**Figure 3**). The top 10 high-yield institutions all come from China (**Table 2**), once again demonstrating that China has invested a great deal of effort in the research on the role of natural products in the intestinal barrier function in this field, and is thus leading in this area. Currently, the cooperation among institutions is still limited to within the country, and cross-national cooperation is still insufficient. Encouraging institutions to take the initiative in seeking cooperation is necessary to promote the development of this field.

**Table 2:** The 10 institutions with high level of cooperation.

No.	Label	Total link strength	Documents	Citations
1	Chinese Academy of Sciences	29	21	719
2	University of Chinese Academy of Sciences	20	11	528
3	Hunan Agricultural University	17	22	768
4	Chinese Academy of Agricultural Sciences	16	19	652
5	China Academy of Chinese Medical Sciences	14	12	153
6	Shanghai University of Traditional Chinese Medicine	14	18	541
7	Beijing University of Chinese Medicine	12	12	130
8	China Agricultural University	12	27	1300
9	Chinese Academy of Medical Sciences and Peking Union Medical College	12	11	187
10	Nanchang University	12	15	949

### 3.4 Research productivity by individual authors

**Figure 4:**
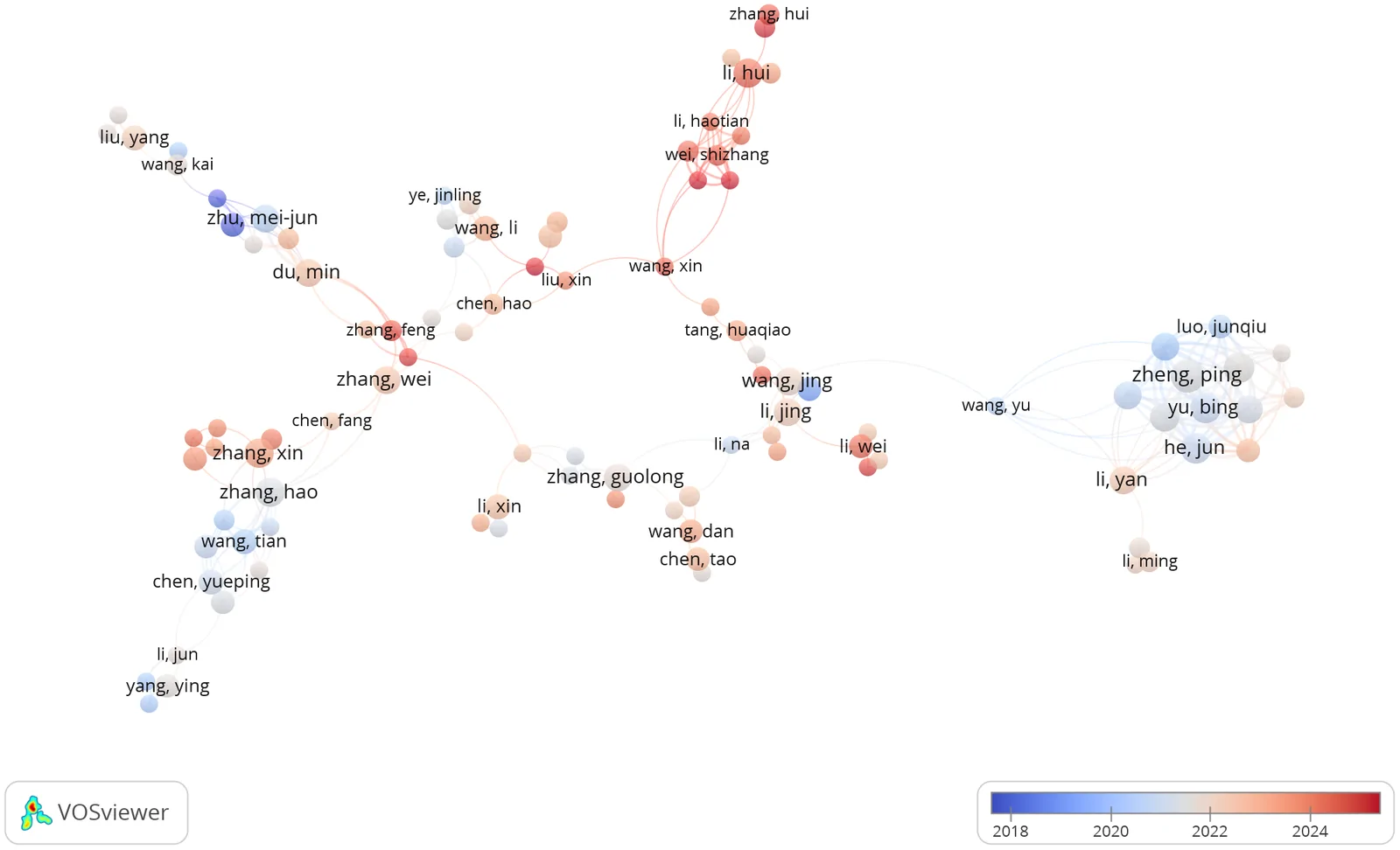
Visual analysis of cooperation between authors. Each node represents an author. The size of the node is positively correlated with the number of published articles. The color of the node is related to the time. The thickness of the lines between the nodes is positively correlated with the frequency of cooperation.

The analysis of the authors is helpful for understanding the representative researchers and core research trends in a field. The H-index can illustrate the significance, importance and wide influence of scientists’ cumulative research contributions (23). A total of 6,346 authors have contributed to the development of this field. **Figure 4** shows the frequency of collaboration among authors, and it can be seen that the scope and frequency of collaboration among authors are small and low. More extensive cooperative relationships among authors should be encouraged (**Table 3**).

**Table 3:** Top 10 authors by publication output.

No.	Name	Total link strength	Documents	Citations
1	zheng, ping	91	10	412
2	he, jun	76	9	411
3	yu, bing	76	9	411
4	huang, zhiqing	70	9	328
5	luo, yuheng	68	8	277
6	galvez, julio	42	8	452
7	zhang, hao	41	8	217
8	zhang, xin	24	8	166
9	li, hui	11	8	56
10	chen, daiwen	60	7	404

### 3.5 Co-cited documents and journals

**Figure 5:**
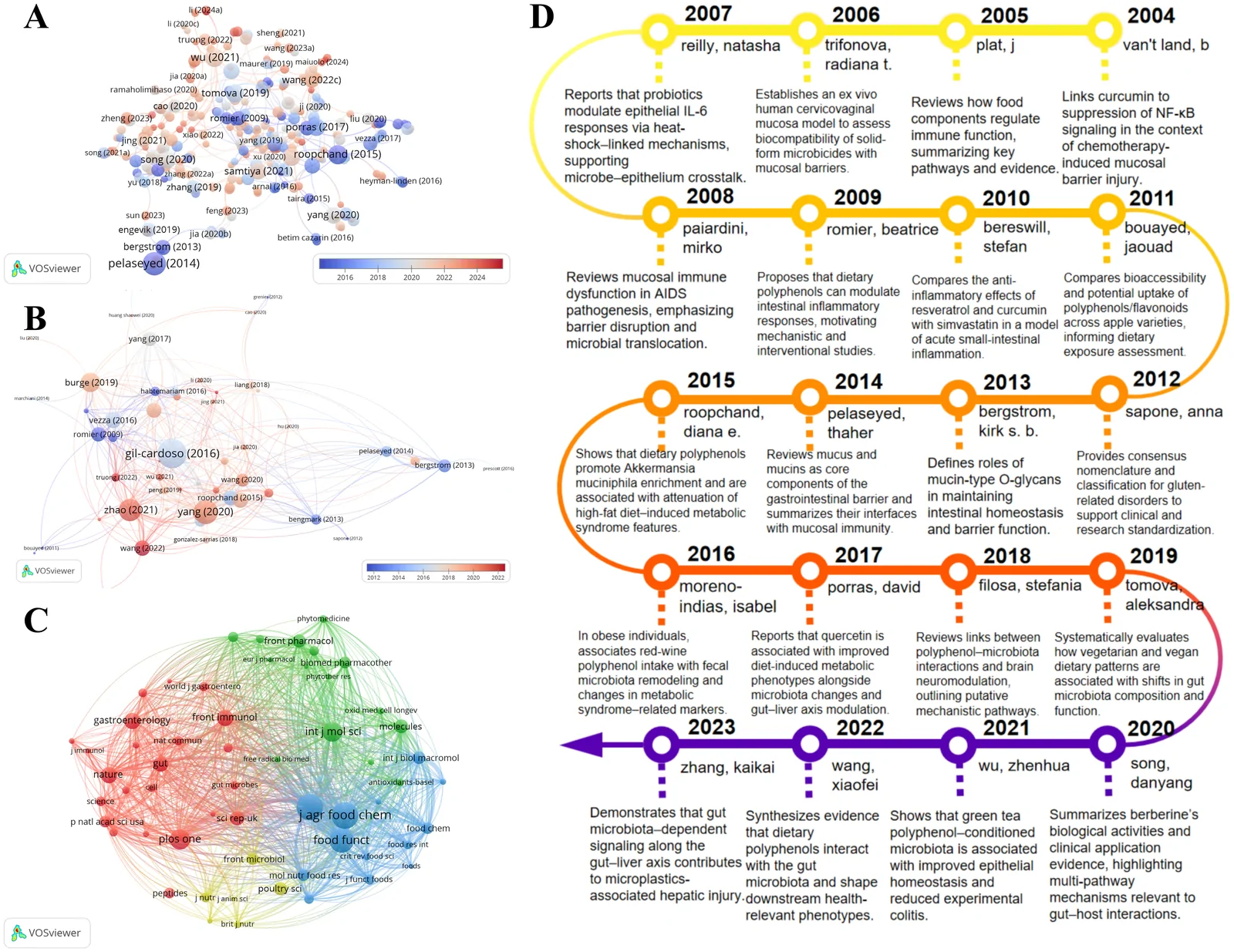
Document analysis. **(A)** Citation analysis of the articles. Each node represents an article, and the size of the node is positively correlated with the number of citations. **(B)** Bibliographic coupling analysis. **(C)** Co-citation of journal. The size of the node is positively correlated with Total Link Strength. Total Link Strength represents the sum of the coupling strength of a certain paper with all other papers in the network. The higher the value, the closer the connection between this paper and other papers in the network. **(D)** Chronological timeline of key articles in natural products in intestinal barrier function research.

The analysis of highly cited articles provides crucial information about specific research fields (**Figures 5A–D**). The number of citations reflects the importance of the articles (**Table 4**). The review by Pelaseyed et al. received the highest number of citations, reaching 998 times. This review summarizes the mucus layer and its close relationship with intestinal cells and goblet cells, as well as the innate and adaptive immune systems, highlighting the focus on the immune system and attracting the research interest of a wide range of researchers. Although the literature published in 2020–2021 was published relatively recently, it has received high citations, indicating that this field is currently in a stage of rapid development.

**Table 4:** Top 10 documents by citation count.

No.	Author	Citations	DOI	REF.
1	pelaseyed (2014)	998	10.1111/imr.12182	(6)
2	sapone (2012)	809	10.1186/1741-7015-10-13	(28)
3	roopchand (2015)	536	10.2337/db14-1916	(29)
4	wu (2021)	502	10.1186/s40168-021-01115-9	(30)
5	tomova (2019)	467	10.3389/fnut.2019.00047	(31)
6	porras (2017)	426	10.1016/j.freeradbiomed.2016.11.037	(32)
7	bolte (2021)	415	10.1136/gutjnl-2020-322670	(33)
8	song (2020)	365	10.1007/s11684-019-0724-6	(34)
9	samtiya (2021)	352	10.3390/foods10040839	(35)
10	yang (2020)	334	10.3390/nu12020381	(36)

A total of 321 journals have published articles in this field. The journal with the highest number of published articles is Food & Function (33 documents), and the journal with the highest citations is Nutrients (1012 times). The number of documents is similar for both, but the Total Link Strength differs by nearly a factor of two. This indicates that Food & Function has a stronger interdisciplinary nature, while Nutrients has greater influence. However, its research topics are relatively cohesive, and the cross-disciplinary connections are not as extensive as those of Food & Function. The top 10 journals ranked by citation count and detailed information are shown in **Table 5**.

**Table 5:** Top 10 journals by total citations.

No.	Label	Total link strength	Documents	Citations	IF (5 years)
1	food & function	103	33	998	6
2	journal of agricultural and food chemistry	86	32	825	6.1
3	journal of functional foods	75	32	495	9.1
4	nutrients	55	31	1012	8.6
5	antioxidants	67	26	747	6.4
6	poultry science	57	26	558	9.4
7	phytomedicine	63	26	498	7.3
8	frontiers in pharmacology	57	24	664	5.2
9	international journal of molecular sciences	50	23	603	5.4
10	frontiers in immunology	57	20	436	10.3

### 3.6 Keyword co-occurrence

**Figure 6:**
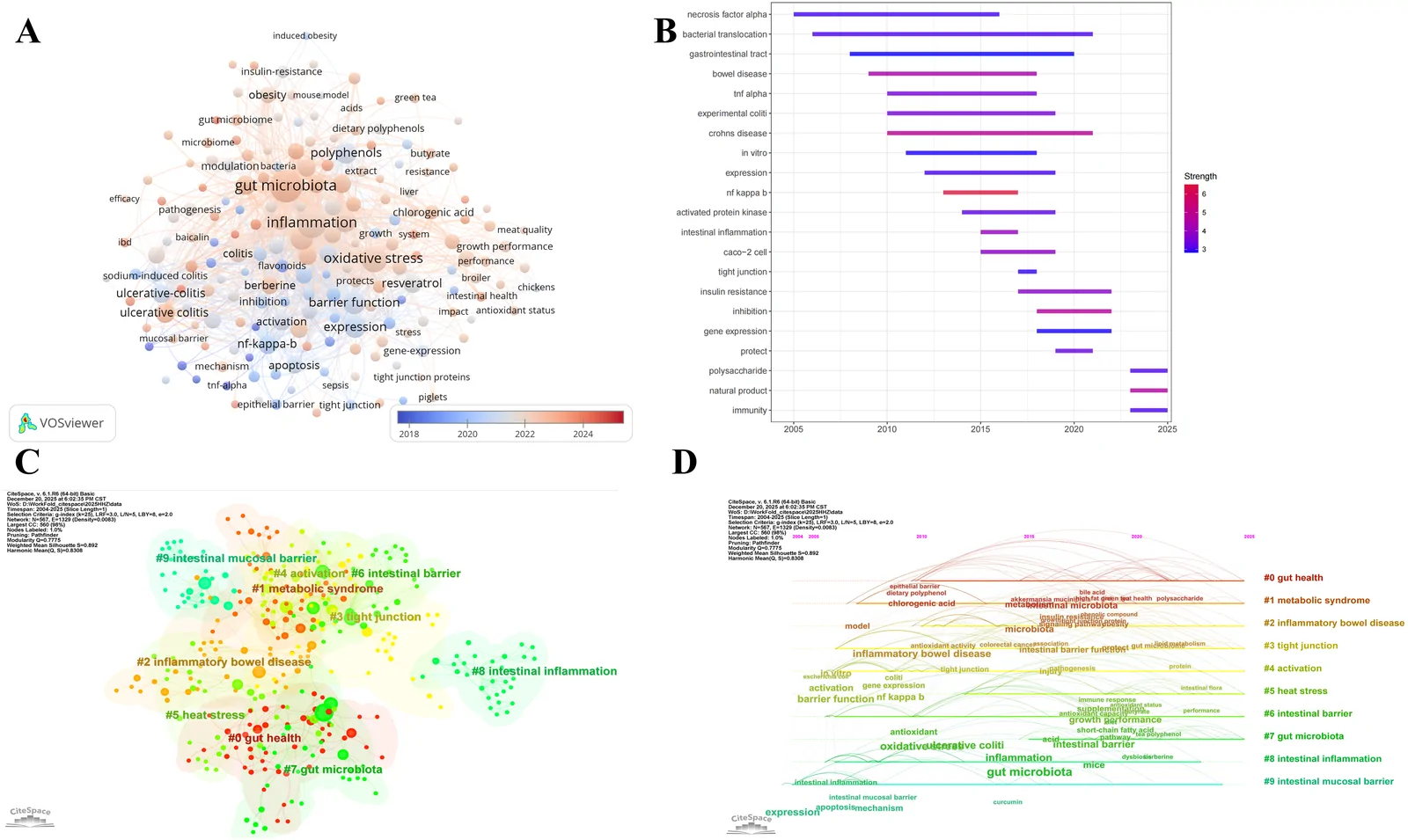
Hotspots and frontiers of keywords. **(A)** Keyword co-occurrence graph. Each node represents a keyword, and the size of the node is proportional to the number of co-occurrences. The two co-cited keywords are connected by a curve, and the color is related to the time when the keyword appears. **(B)** Top 20 keywords with the strongest citation burst. Color segment represents the start year, end year and burst duration of the first occurrence of keywords, and color is positively correlated with the intensity of burst. **(C)** Cluster analysis of keywords. Each cluster consists of several closely related keywords, and the size of the cluster tag (#) sequence number is displayed as # 0, # 1, # 2, etc. **(D)** Keywords timeline diagram.

**Figure 6A** presents the co-occurrence situation of keywords (**Table 6**). “gut microbiota” ranks first with the highest link strength and occurrences (2687, 318), indicating that the composition, proportion, and quantity of the gut microbiota play a core role in the regulation of the intestinal barrier function. Following closely are “inflammation” (2368, 258) and “oxidative stress” (1726, 185), which are important mechanisms for the progression of many diseases. “nf-kappa-b” (978, 95) is a key inflammatory factor and also occupies an important position in the intestinal barrier function. Ulcerative colitis is the most studied disease type, indicating that the intestinal barrier function plays an important role in the development of this disease. Polyphenols (1040, 112) are the substances with the highest occurrences, indicating that polyphenols are the most extensively studied natural substance category. Resveratrol (868, 77) has a link strength far exceeding its frequency (ratio 11.27), suggesting that resveratrol is a hub substance connecting different levels and pathways, possibly simultaneously correlating with different mechanisms such as microbial and inflammatory signaling pathways. “mice”, “*in-vitro*”, and “cells” indicate that *in vitro* experiments remain the main method of research.

**Table 6:** Top 20 keywords by co-occurrenc.

No.	Label	Total link strength	Occurrences
1	gut microbiota	2687	318
2	inflammation	2368	258
3	oxidative stress	1726	185
4	intestinal barrier	1046	131
5	barrier function	1211	128
6	expression	1104	119
7	microbiota	1086	118
8	polyphenols	1040	112
9	mice	721	86
10	ulcerative colitis	671	86
11	nf-kappa-b	978	85
12	resveratrol	868	77
13	ulcerative-colitis	701	77
14	colitis	637	75
15	activation	699	74
16	health	639	74
17	barrier	601	73
18	berberine	565	70
19	*in-vitro*	642	69
20	cells	572	68

**Figure 6B** is the keyword burst graph of natural products in the research of intestinal barrier function, showing the evolution of research hotspots in this field from 2004 to 2025. In the early stage of the research (before 2010), the burst intensity of keywords related to basic molecular mechanisms was higher, such as “necrosis factor alpha” and “tnf alpha”, indicating that the research focus at this stage was on understanding how natural products affect the intestinal barrier function by regulating key signaling pathways. In the middle stage (2010-2020), research began to focus on the structure and function of the intestinal barrier, such as “tight junction”, “caco−2 cell”, “*in vitro*”, “gene expression”, “inhibition”, etc., the appearance of these keywords indicates that this stage integrated the molecular mechanisms with the barrier structure and function for analysis. “bacterial translocation” has the longest burst duration (2006-2021), indicating that the gut microbiota is the core content of this field of research. The burst intensity of “nf kappa b” is the highest, indicating that the “intestinal inflammation” related to this pathway is an important mechanism for the regulation of the intestinal barrier function. In 2025, keywords such as “natural product” and “immunity” will continue to burst, indicating that the current focus on natural products as a representative of immune regulation and barrier function regulation is a hot topic in this field.

The keyword clustering graph generated based on the co-occurrence network is shown in **Figure 6C**, and the 10 clusters formed represent the most prominent themes in this field (**Figure 6D**; **Table 7**). Overall, it is presented as four aspects: barrier structure/function (#3 tight junction, #4 activation, #6 intestinal barrier, #9 intestinal mucosal barrier), inflammatory response (#2 IBD, #5 heat stress, #8 intestinal inflammation), metabolic abnormalities (#1 metabolic syndrome), and microbiota regulation (#7 gut microbiota, #0 gut health). Further analysis of the clustering (**Figure 6**) shows that the gut microbiome (#0, #7), intestinal inflammation (#2, #8), and intestinal barrier function (#3, #6, #9) have always been hot research topics.

**Table 7:** Keywords clusters tag information.

ID	Size	Silhouette	Label
0	48	0.794	gut microbiota; colonic barrier function; m1 macrophage; intestinal barrier function; atopic dermatitis; ulcerative colitis; fecal microbiota transplantation; short-chain fatty acids; dehydrocostus lactone; berberine hydrochloride
1	41	0.827	gut microbiota; mediterranean diet; psoriatic arthritis; omega-3 fatty acids; ankylosing spondylitis; metabolic syndrome; oxidative stress; insulin resistance; fructose-rich diet; chinese herbal formulation
2	41	0.908	gut microbiota; metabolic disorder; oxidative stress; natural products; nrf2 pathway; inflammatory bowel disease; intestinal stem cell; high lipid metabolism; a methylation; cholinergic system
3	38	0.902	tight junction; reporter assay; chemical modulator; chemical profiling; chronic colitis; intestinal barrier; antioxidant capacity; intestinal epithelial cells; hydrolysis degree; intestinal barrier function
4	36	0.93	ulcerative colitis; intestinal microbiota; goblet cells; butyryl-coa transferase gene; lupinus albus; intestinal barrier; antioxidant capacity; intestinal dismicrobism; phytic acid; hydrolysis degree
5	35	0.902	growth performance; weaned piglets; salmonella typhimurium; antimicrobial alternatives; olive oil by-products; heat stress; intestinal barrier; antioxidant capacity; growing chickens; chlorogenic acid
6	35	0.857	intestinal barrier; network pharmacology; laying hens; phytic acid; scutellaria baicalensis; gut microbiota; inflammatory bowel disease; inflammatory signaling pathways; intestinal physical barrier; chinese medicine
7	32	0.856	gut microbiota; amino acid metabolism; colitis-associated colorectal cancer; wnt signaling pathway; dss-inducedchronic colitis; intestinal barrier; network pharmacology; dss-inducedchronic colitis; supplementation; atopic dermatitis
8	31	0.969	intestinal inflammation; ulcerative colitis; sodium-induced colitis; dextran sulfate; intestinal mucosal barrier; organic selenium; aquaculture species; gastrointestinal tract; mannan oligosaccharide; natural defence
9	29	0.975	intestinal mucosal barrier; oxidative stress; disease prevention; natural flavonoids; chlorogenic acid; ulcerative colitis; intestinal epithelial cells; nanoparticle-mediated drug delivery systems; natural bioactive compounds; programmed cell death

## 4 Discussion

### 4.1Summary of findings

In the present study, we systematically searched the WoSCC and PubMed for publications related to natural products and intestinal barrier function published between January 1, 2004, and November 6, 2025. Following a rigorous screening process, a total of 1,679 publications were included for comprehensive bibliometric and visual analyses. Overall, the annual number of publications increased steadily throughout the period from 2004 to 2025, with a marked acceleration observed after 2020. This inflection point may be attributed to the rapid adoption of high-throughput sequencing technologies, the declining costs of multi-omics approaches, and the widespread application of causal validation tools, such as germ-free mouse models, during this period (37). The maturation of these technologies and the reduction in technical and economic barriers have provided researchers with the essential methodological foundation to elucidate the mechanisms by which natural products regulate intestinal barrier function from the perspective of gut microbiota–host barrier interactions. Collectively, these findings suggest that technological innovation has been a major driving force behind the rapid expansion of this research field. Against this background, increasing attention has been directed toward the protective and restorative effects of natural products on the intestinal barrier, with their underlying mechanisms of action emerging as a major research hotspot and frontier.

A total of 63 countries have contributed to research in this field, although scientific output exhibits pronounced national and regional clustering. China ranked first with 682 publications and 15,987 citations (**Table 1**), and all of the top 10 most productive institutions were from China (**Table 2**). However, analysis of the institutional collaboration network revealed that collaborative links were predominantly domestic, whereas international collaborations remained relatively limited. This finding suggests that the field of natural products and intestinal barrier function has not yet established closely integrated international research networks. The author collaboration network further supports this observation: collaborations among the 6,346 identified researchers were largely confined to within individual institutions or domestic research teams, and extensive cross-national and interdisciplinary author networks have yet to emerge. The current collaboration pattern remains relatively fragmented, with limited collaborative intensity, thereby restricting the integration of research resources and complementary expertise across different natural products. Broader international and interdisciplinary collaborations may therefore accelerate the transition of this field from descriptive studies toward mechanistic investigations, translational research, and the integration of clinical resources for practical applications.

At the journal level, Nutrients ranked first with 1,012 citations, highlighting its leading role in synthesizing evidence and disseminating knowledge in this field. Food & Function, with 33 publications, was the most productive journal, and its total link strength (103) was substantially higher than those of journals with a comparable publication outputs. This contrast indicates that Food & Function serves as a more specialized platform for research on food-derived natural compounds and intestinal barrier function, while demonstrating stronger interdisciplinary characteristics by bridging chemistry, biology, and medicine. Bibliometric analysis further showed that publications from 2020–2021 had already accumulated relatively high citation counts despite their recent publication dates, whereas publications from 2020–2022 exhibited strong bibliographic coupling, indicating that research in this field is rapidly converging around several emerging topics and forming new knowledge clusters. These findings suggest that the field is currently undergoing a critical stage of rapid knowledge accumulation and the establishment of new research paradigms.

The keyword co-occurrence network provides direct evidence for the underlying knowledge structure of this field. “Gut microbiota” occupied the central position, with the highest total link strength (2,687) and occurrence frequency (318), and, together with mechanistic keywords such as “inflammation” (2,368) and “oxidative stress” (1,726), constituted the core knowledge framework of the field. Meanwhile, “NF-κB” (978) functioned as a key signaling node linking microbial signals with inflammatory responses. Collectively, these findings indicate that the “gut microbiota–inflammation–oxidative stress” axis represents the central mechanistic framework through which natural products regulate intestinal barrier function. Among individual natural products, resveratrol exhibited the highest ratio of total link strength to occurrence frequency (11.27). This pattern suggests that resveratrol is highly connected with multiple research topics rather than within a single mechanistic context. Its co-occurrence with keywords related to gut microbiota, inflammation, oxidative stress, and intestinal barrier function suggests that it has become a representative model compound for exploring the interconnected mechanisms underlying intestinal barrier regulation, reflecting its broad research relevance.

Keyword burst analysis further illustrates the evolution of research priorities in this field. During the early stage (before 2010), burst keywords were dominated by classical inflammatory mediators, including “necrosis factor alpha” and “TNF alpha”, reflecting a primary focus on the direct regulation of key signaling pathways by natural products. During the intermediate stage (2010–2020), research shifted toward barrier structure-related topics, including “tight junction”, “Caco-2 cell”, and “*in vitro*”, indicating an increasing integration of molecular mechanisms with intestinal barrier function. In recent years, continuous bursts of keywords such as “natural product” and “immunity” suggest that immune regulation and its coupling with intestinal barrier homeostasis have become the most active frontier of research in this field.

However, the high-frequency occurrence of keywords such as “mice”, “*in vitro*”, and “cells” highlights an important research gap: current evidence is still predominantly derived from animal and cell-based studies, whereas clinical evidence remains relatively limited. The predominance of keywords such as “mice”, “*in vitro*”, and “cells” indicates that mechanistic investigations based on preclinical models continue to dominate this field, whereas clinical validation remains relatively scarce. Bibliometric analysis shows that the most frequently investigated compounds, including berberine, curcumin, and resveratrol, are predominantly studied as isolated molecules under controlled experimental conditions. However, in clinical practice, these compounds are usually administered as components of botanical extracts or traditional herbal prescriptions rather than as purified monomers. Such reduction from chemically complex formulations to single compounds inevitably simplifies the pharmacological context and may eliminate synergistic interactions, buffering effects, and multi-component regulation that are considered essential characteristics of traditional herbal medicines.

Furthermore, the dominant mechanistic framework identified in the present analysis reflects a modern target-oriented biomedical paradigm, including NF-κB, Nrf2, oxidative stress, and tight junction signaling. In contrast, traditional clinical application of herbal medicines is generally guided by syndrome differentiation and formula compatibility, in which therapeutic decisions are based on the correspondence between herbal formulas and patient patterns rather than isolated compound–target interactions. Consequently, mechanistic evidence obtained from simplified experimental systems may be difficult to translate directly into clinical prescription strategies. Future studies may therefore benefit from integrative research strategies that preserve the complexity of botanical extracts or classical herbal formulas while incorporating biomarker-based endpoints together with syndrome-oriented clinical outcome measures. Although this field is undergoing a period of rapid development, the translation of mechanistic evidence into clinical applications remains insufficient and warrants further investigation.

Based on the above bibliometric findings, the subsequent discussion will focus on the gut microbiota, microbial metabolites, and signaling pathways. Specifically, we will summarize the key mechanisms by which natural products regulate intestinal barrier function through the NF-κB and Nrf2 signaling pathways in the context of gut microbiota-mediated barrier regulation. In addition, we will discuss the potential and challenges of this emerging research field and further explore immunity, one of the current research frontiers, as a priority direction for future translational research and clinical applications.

### 4.2 The regulation of gut microbiota in intestinal barrier function

Life Science Identifiers (LSIDs) for ZOOBANK registered names or nomenclatural acts should be listed in the manuscript before the keywords with the following format:

The central role of the gut microbiota is strongly supported by the keyword co-occurrence network, in which “gut microbiota” exhibited the highest total link strength (2,687) and occurrence frequency (318). Accumulating evidence has established the gut microbiota as an upstream regulator of intestinal barrier homeostasis. Structurally, the gut microbiota and the intestinal barrier are intimately interconnected and can be organized into three functional layers extending from the intestinal lumen toward the underlying tissues. The outermost layer is the mucus layer, which contains commensal microorganisms, antimicrobial peptides (AMPs), and secretory immunoglobulin A (sIgA). The middle layer consists of a specialized monolayer of intestinal epithelial cells that serves as the core physical barrier. The innermost layer is the lamina propria, which contains both innate and adaptive immune cells, including T cells, B cells, macrophages, and dendritic cells (11, 12).

When dysbiosis is induced by diet, medications, or disease, alterations in microbial metabolites, disruption of the mucus barrier, breakdown of tight junctions, and upregulation of pro-inflammatory signaling frequently occur in a coordinated manner. This cascade of events increases intestinal permeability, leading to a leaky gut phenotype that facilitates the translocation of microbiota-associated molecular components into the lamina propria. The subsequent activation of resident immune cells triggers mucosal and systemic low-grade inflammation, ultimately contributing to the initiation and progression of a wide range of diseases.

#### 4.2.1 Regulation of structural Integrity

The intestinal epithelial monolayer, together with the tight junctions (TJs) between adjacent epithelial cells, constitutes the physical barrier of the intestine. Tight junctions are composed of proteins including Claudins, Occludin, and zonula occludens-1 (ZO-1). Among these, members of the Claudin family form charge-selective ion pores that determine barrier permeability and regulate the selective transport of molecules through both transcellular and paracellular pathways (38). External to this epithelial barrier, the mucus layer provides an additional level of protection by offering segment-specific physical separation and chemical defense throughout different regions of the intestine (39). The gut microbiota plays a pivotal role in maintaining intestinal barrier function. Microbiota-derived metabolites, particularly short-chain fatty acids (SCFAs) and bile acids, activate host epithelial receptors such as GPR109A and farnesoid X receptor (FXR), thereby regulating the expression and localization of tight junction proteins, including Claudins and Occludin, while promoting mucus secretion to enhance intestinal barrier integrity (40). As the primary energy source for colonic epithelial cells, butyrate is produced through microbial fermentation of dietary fiber and enhances the expression of tight junction proteins by activating AMPK and MLCK/MLC2 signaling pathways, thereby promoting epithelial cell proliferation and barrier repair (41–43). Other SCFAs, such as propionate and acetate, similarly contribute to the restoration of intestinal epithelial barrier integrity through related mechanisms (44). Conversely, gut microbial dysbiosis can directly damage intestinal epithelial cells, impair barrier integrity, and increase intestinal permeability (45, 46). Supplementation with beneficial gut microorganisms, such as Lactobacillus sp. BP121, has been shown to increase the relative abundance of butyrate-producing bacteria, upregulate tight junction protein expression, and restore impaired intestinal barrier function (47).

Tight junctions between intestinal epithelial cells are the structural basis of intestinal barrier function, and their integrity depends on the coordinated assembly of transmembrane proteins, including Claudins and Occludin, with scaffold proteins such as ZO-1 (48). Transmembrane mucins, including MUC1 and MUC3, function as barrier effector proteins and are expressed on the apical surface and lateral membranes of intestinal epithelial cells, where they prevent direct contact between bacteria and the epithelial surface (49). Both *in vitro* and *in vivo* studies have demonstrated that beneficial microorganisms promote mucin synthesis and secretion (50–52), thereby enhancing the structural integrity and functional stability of the epithelial barrier (53). During tight junction assembly, scaffold proteins ZO-1, ZO-2, and ZO-3 recruit and anchor transmembrane tight junction proteins through protein interaction domains such as PDZ and SH3, thereby forming a stable junctional complex (54–57). Loss of ZO-1 prevents the recruitment of Claudins to the tight junction complex, resulting in increased intestinal permeability and compromised barrier function (58, 59).

A growing body of evidence has demonstrated that diverse natural products protect and restore intestinal barrier integrity through distinct yet often convergent mechanisms. Quercetin, a dietary flavonoid widely distributed in plant-based foods, promotes cytoskeletal organization and enhances the expression of multiple tight junction proteins, including ZO-1, Occludin, and Claudins (60). 6-Gingerol restores intestinal barrier function by upregulating Claudin-4, Occludin, and ZO-1 expression while reducing intestinal permeability (61). Curcumin has been shown to enhance the expression of ZO-1 and Claudin-1 in human intestinal epithelial Caco-2 cells, thereby restoring epithelial architecture, improving barrier function, reducing paracellular permeability, and protecting the intestine against ischemia/reperfusion-induced injury (14, 62, 63).

The intestinal mucus layer is a gel-like barrier covering the surface of the intestinal epithelium and plays a central protective role in maintaining intestinal homeostasis by preventing mechanical injury, chemical irritation, and biological insults (64, 65). Covering the luminal surface of the intestinal epithelium, the mucus layer effectively shields epithelial cells from toxic substances, digestive enzymes, and bacteria (66). Once the mucus layer is compromised, opportunistic pathogens and pathogenic microorganisms can readily reach the intestinal epithelium, thereby triggering infection and inflammation (67). The gut microbiota plays a crucial coordinating role in maintaining mucus layer homeostasis. By promoting mucin secretion from goblet cells, the gut microbiota preserves the thickness and quality of the mucus layer, thereby effectively preventing direct bacterial contact with epithelial cells (68, 69). Beneficial microorganisms, such as Lactobacillus and Bifidobacterium, also occupy binding sites within the mucus layer, thereby inhibiting the degradation and penetration of mucus components by pathogenic bacteria (70).

Mechanistic studies have demonstrated that several natural products exert protective effects on the mucus barrier. Curcumin has been shown to promote the secretion of acidic intestinal mucus (71), improve the structural integrity and immune function of the intestinal mucosal barrier (72), and effectively maintain the integrity of the mucus layer (73). Polysaccharides derived from Abelmoschus manihot enhance the intestinal mucus barrier and alleviate intestinal inflammation by modulating the abundance of Akkermansia muciniphila. These polysaccharides stimulate goblet cells to secrete MUC2, thereby preserving mucus layer integrity and preventing pathogenic microorganisms from penetrating the mucus layer and directly contacting intestinal epithelial cells (74).

#### 4.2.2 Chemical barrier regulation

The intestinal chemical barrier complements the mechanical barrier, and together they function synergistically to maintain intestinal microecological homeostasis while serving as a critical interface between innate immunity and the gut microbiota (75–77). This barrier consists of a defensive system composed of gut microbiota-derived metabolites, enzymes, and host-secreted molecules, including mucins and antimicrobial peptides. Mucin 2 (MUC2), secreted by goblet cells, represents the principal component of the intestinal chemical barrier in both mice and humans (78, 79). In addition to mucins, antimicrobial peptides, such as defensins and lysozyme, together with secretory immunoglobulin A (sIgA), enhance the protective function of the chemical barrier by inactivating or suppressing pathogenic microorganisms and restricting the overgrowth of commensal bacteria (2).

Short-chain fatty acids (SCFAs), generated through microbial fermentation of dietary fiber, are key components of the intestinal chemical barrier. SCFAs promote mucin synthesis and secretion by goblet cells (80), selectively upregulate the expression of MUC genes in intestinal goblet cells (81), and stimulate Paneth cells in the small intestine to secrete α-defensins (82), thereby strengthening the intestinal chemical barrier through multiple mechanisms and targets. SCFAs also lower the luminal pH of the intestine (83), and under acidic conditions (pH 5.5), high concentrations of SCFAs inhibit the growth and proliferation of pathogenic bacteria (84). In addition, microbiota-derived tryptophan metabolites, including indole and its derivatives, promote interleukin-22 (IL-22) secretion through activation of the aryl hydrocarbon receptor (AhR), thereby inducing antimicrobial peptide production. This pathway has emerged as a major focus of recent studies on the intestinal chemical barrier (85).

The gut microbiota also contributes to the chemical barrier by producing antimicrobial proteinaceous particles that restrict the excessive expansion of pathogens during intestinal inflammation (86). Paneth cells and intestinal epithelial cells secrete a variety of antimicrobial peptides that prevent pathogenic microorganisms from colonizing the epithelial surface (87, 88). Furthermore, the gut microbiota converts primary bile acids into secondary bile acids, which reshape the microbial community through their antimicrobial activity and indirectly reduce the abundance of endotoxin-producing bacteria (89). Segmented filamentous bacteria (SFB) induce germinal center activation within Peyer’s patches (PPs) and promote immunoglobulin A (IgA) class switching, making them among the most potent inducers of sIgA production (90). By binding to pathogens and toxins, sIgA traps these agents within the mucus layer through a non-inflammatory mechanism known as immune exclusion and facilitates their removal by intestinal peristalsis, thereby preventing their adhesion to the intestinal epithelium (91). In addition, sIgA suppresses bacterial virulence and is therefore regarded as a central effector molecule of the intestinal chemical barrier, integrating both immune recognition and chemical defense functions (92, 93).

### 4.2.3Immune barrier regulation

The intestinal immune barrier consists of immune cells distributed within the gut-associated lymphoid tissue, including macrophages, T lymphocytes, innate lymphoid cells, and lamina propria lymphocytes, as well as Peyer’s patches, mesenteric lymph nodes, and the immune effector molecules secreted into the intestinal lumen (94).

In addition to secreting mucus, goblet cells participate in mucosal immune regulation through the release of immunomodulatory factors. Dysfunction of the mucus barrier and abnormalities in goblet cell number or function are closely associated with intestinal inflammation. In some cases, impairment of the mucus system even precedes the clinical manifestation of inflammation, suggesting that goblet cells play an initiating role in the pathogenesis of intestinal diseases (39). Intestinal epithelial cells recognize microbe-associated molecular patterns (MAMPs) through pattern recognition receptors (PRRs) and transmit these signals to mucosal immune cells, thereby coordinating immune tolerance toward commensal microorganisms with protective immune responses against invading pathogens (5).

Gut microbiota-derived metabolites serve as upstream signaling molecules involved in regulating the intestinal immune barrier. Short-chain fatty acids (SCFAs) exert immunomodulatory effects through multiple mechanisms, including activation of G protein-coupled receptors GPR41, GPR43, and GPR109A, as well as inhibition of histone deacetylases (HDACs). These mechanisms promote immune tolerance and enhance intestinal secretory immunoglobulin A (sIgA) responses (95, 96). SCFAs primarily promote the expression of hypoxia-inducible factor-α (HIF-α) and the aryl hydrocarbon receptor (AhR) in CD4^+^ T cells and innate lymphoid cells (ILCs) through GPR41 activation and HDAC inhibition, thereby stimulating the production of interleukin-22 (IL-22). IL-22 subsequently promotes epithelial repair, induces antimicrobial peptide expression, and strengthens mucosal barrier function (97). In addition, metabolic signals such as SCFAs facilitate IgA coating of intestinal microorganisms without provoking excessive inflammatory responses, thereby limiting bacterial adhesion and invasion (98).

The gut microbiota also plays an essential role in bile acid metabolism. Following secretion into the intestine, conjugated primary bile acids synthesized by the liver are deconjugated by microbial bile salt hydrolases to generate unconjugated primary bile acids, which are subsequently converted into various secondary bile acids, including deoxycholic acid and lithocholic acid, through reactions such as 7α-dehydroxylation. Secondary bile acids activate Takeda G protein-coupled receptor 5 (TGR5) to regulate intestinal stem cell-mediated epithelial renewal, while enhancing tight junction integrity through the farnesoid X receptor (FXR) and TGR5 signaling pathways. Activation of FXR suppresses NF-κB-mediated transcription of pro-inflammatory cytokines, whereas TGR5 activation inhibits inflammasome assembly. Together, these mechanisms elevate the inflammatory threshold of the intestine, modulate mucosal immune responses, and maintain intestinal barrier homeostasis (99). After reaching the colon, polyphenolic natural products are biotransformed by the gut microbiota into metabolites with greater bioavailability and enhanced biological activity. These metabolites selectively modulate the composition and metabolic profile of the gut microbiota, thereby alleviating intestinal inflammation and improving intestinal barrier function (100, 101).

#### 4.2.4 Microbial barrier

The gut microbiota suppresses pathogen proliferation through multiple mechanisms, including competition for nutrients, ecological niche exclusion, and induction of host immune responses. Collectively, these mechanisms are referred to as colonization resistance (102, 103). Colonization resistance describes the ability of the commensal microbiota to prevent pathogen colonization and excessive growth (104), thereby protecting the host against pathogenic infection. Fundamentally, it represents an intestinal defense mechanism provided by the commensal microbiota, in which commensal microorganisms directly compete with pathogens for ecological niches and nutrient resources. For example, Bifidobacterium spp. produce acetate, which enhances epithelial-mediated intestinal defense and protects the host against lethal enteric infections (15). The resident intestinal microbiota plays a central role in preventing pathogen colonization and subsequent intestinal infection (15, 105). Commensal gut microorganisms enhance resistance to pathogenic colonization by directly competing with pathogens for available nutrient sources, thereby limiting their colonization and dissemination within the intestine (103). In addition to suppressing the growth of intestinal pathogens, the gut microbiota also plays a critical role in eliminating invading bacteria.

Accumulating evidence indicates that natural products can reinforce colonization resistance by modulating the gut microbiota. Cranberry proanthocyanidins (PACs) effectively inhibit the adhesion of pathogenic Escherichia coli to the intestinal epithelium through a non-bactericidal anti-adhesion mechanism, thereby reducing bacterial shedding *in vivo*. In experimental models, cranberry PACs significantly reduced fecal shedding of pathogenic E. coli, shortened the duration of bacterial excretion, alleviated the severity of diarrhea, and decreased serum pathogen-specific antibody levels (106). Ursolic acid has also been shown to ameliorate intestinal injury and microbial dysbiosis in mice by remodeling the gut microbial community, increasing the abundance of short-chain fatty acid-producing bacteria, and enhancing intestinal barrier function (107).

The gut microbiota functions not only as an upstream regulator of intestinal barrier integrity but also as the primary source of signaling input for the intestinal immune barrier. Consistent with the trends identified by the keyword burst analysis in the present study, future research is expected to move beyond the simple improvement of intestinal barrier function toward elucidating the interactions between immune networks and barrier homeostasis. In particular, increasing attention will be directed toward understanding the mechanisms by which natural products regulate intestinal diseases through the integrated “gut microbiota–metabolites–signaling pathways–barrier function” axis.

### 4.3 Pathway-centric mechanisms of natural products in intestinal barrier regulation

In the keyword co-occurrence network, “NF-κB” (total link strength = 978) and “oxidative stress” (total link strength = 1,726) represent the central nodes of the inflammatory and oxidative stress modules, respectively, whereas “polyphenols” (occurrence frequency = 112) and “resveratrol” (total link strength-to-occurrence ratio = 11.27) highlight the prominent role of natural products within this signaling network. The protective effects of natural products on the intestinal barrier generally follow a sequential mechanism characterized by “gut microbiota remodeling–signal transduction–barrier response.” Specifically, natural products first remodel the gut microbial community and its metabolic profile, after which microbiota-derived metabolites activate receptor-mediated signaling and inflammatory/antioxidant hub pathways. These signals are subsequently transmitted to the chemical and immune barriers, ultimately resulting in the stabilization of tight junction protein expression and the promotion of epithelial regeneration and repair. This mechanistic framework has been validated for several natural products. For example, butyrate enhances intestinal barrier function by activating AMP-activated protein kinase (AMPK) in Caco-2 cell monolayers, thereby promoting tight junction assembly (108). Similarly, flavonoids exert barrier-protective effects by activating bile acid receptors and regulating the expression of tight junction proteins (109).

#### 4.3.1 TLR4/TNF–NF-κB–MLCK axis links immune inflammation to tight junction leak

NF-κB-related signaling pathways exhibited both high co-occurrence and strong burst intensity in the present study, suggesting that NF-κB is a central molecular target through which natural products regulate intestinal barrier function. NF-κB is a master transcription factor governing pro-inflammatory responses. Its excessive activation induces the production of numerous pro-inflammatory cytokines, including TNF-α, IL-1β, and IL-6, which suppress the synthesis of tight junction proteins such as ZO-1 and Occludin, thereby reducing tight junction protein expression and increasing intestinal epithelial permeability (110). TNF-α further activates MLCK gene transcription through the NIK/IKKα/NF-κB p50/p65 signaling axis, disrupting tight junction architecture and increasing paracellular epithelial permeability, thereby translating immune-inflammatory signals into physical barrier damage (111). Collectively, the “inflammation–signaling–barrier” axis represents a critical mechanistic framework underlying the protective effects of natural products on the intestinal barrier.

A wide range of natural products exhibit potent anti-inflammatory activities, primarily by suppressing pro-inflammatory signaling pathways such as NF-κB, thereby reducing the production of inflammatory mediators. Resveratrol alleviates intestinal barrier injury through modulation of the TLR4/MyD88/NF-κB signaling pathway (112). In dextran sulfate sodium (DSS)-induced colitis models, resveratrol markedly suppressed the production of pro-inflammatory cytokines, increased transepithelial electrical resistance, and enhanced the expression of the tight junction proteins ZO-1 and Occludin (113).

(-)-Epigallocatechin-3-gallate (EGCG) suppresses MAPK and NF-κB signaling in macrophages and dendritic cells, thereby reducing lipopolysaccharide (LPS)-induced immune cell maturation and inflammatory cytokine release. Low-dose EGCG has been shown to significantly ameliorate experimental colitis in mice by reducing inflammatory cell infiltration, decreasing the production of pro-inflammatory cytokines, and improving intestinal permeability. Curcumin markedly prevents TNF-α-induced tight junction disruption and inhibits the increase in barrier permeability resulting from NF-κB activation (14, 63). In addition, curcumin promotes the polarization of macrophages and other immune cells toward an anti-inflammatory phenotype, thereby reducing mucosal tissue injury. Berberine suppresses the excessive activation of innate immune pathways, including the NLRP3 inflammasome, and inhibits NF-κB-mediated pro-inflammatory responses in macrophages and neutrophils (114). Collectively, these anti-inflammatory mechanisms enable natural products to attenuate immune-mediated intestinal barrier injury while coordinately maintaining immune exclusion capacity and intestinal barrier homeostasis.

#### 4.3.2 Keap1–Nrf2/HO-1 redox gating promotes junctional restoration and mucosal repair

Bibliometric analysis revealed that oxidative stress-related topics exhibited a pronounced burst pattern in the present study, with burst intensity increasing markedly in recent years, suggesting that the Nrf2 signaling axis is a highly probable molecular hub through which natural products improve intestinal barrier function. Nuclear factor erythroid 2-related factor 2 (Nrf2) is the master regulator of the cellular antioxidant response. Upon activation, Nrf2 translocates into the nucleus and binds to antioxidant response elements (AREs), thereby inducing the expression of antioxidant and detoxification enzymes, including heme oxygenase-1 (HO-1), NAD(P)H:quinone oxidoreductase 1 (NQO1), and glutathione (GSH) synthesis-related enzymes. These responses effectively eliminate excessive reactive oxygen species (ROS) and attenuate oxidative damage to intestinal epithelial cells and goblet cells (115, 116). Previous studies have demonstrated that Nrf2 negatively regulates the NF-κB signaling pathway, enhances tight junction integrity, and promotes epithelial regeneration and mucosal repair through its antioxidant and cytoprotective activities, thereby preserving epithelial barrier integrity and mucosal healing capacity (117). Conversely, impaired Nrf2 activity results in excessive oxidative stress and dysregulated inflammatory responses, manifested by increased intestinal permeability, aggravated mucosal injury, and exaggerated immune activation (118). Therefore, the Nrf2 signaling pathway is indispensable for protecting the mucus layer and intestinal epithelial cells against oxidative injury and for maintaining intestinal barrier homeostasis.

A growing body of evidence has demonstrated that numerous natural products exert barrier-protective effects through activation of the Nrf2 signaling pathway. Resveratrol upregulates the expression of Nrf2 and its downstream target HO-1, thereby scavenging reactive oxygen species, attenuating oxidative damage to intestinal epithelial cells, and ultimately improving intestinal barrier function (119, 120). Chlorogenic acid (CGA) alleviates heat stress (HS)-induced intestinal oxidative injury through activation of the Nrf2 signaling pathway while preserving barrier integrity (121). Urolithin A, a gut microbiota-derived metabolite, activates signaling pathways including the AhR-Nrf2 axis, thereby inducing the expression of tight junction proteins such as Claudin, Occludin, and ZO-1, protecting intestinal epithelial barrier integrity, and enhancing barrier function (117). By activating the Nrf2-dependent antioxidant signaling axis, natural products effectively attenuate oxidative stress-driven inflammatory cascades that damage the mucus layer and intestinal epithelial cells, thereby contributing to the maintenance of mucosal barrier homeostasis (122).

#### 4.3.3 Programmed cell death and barrier: direct destruction of the barrier

Restoration of intestinal barrier function ultimately depends on epithelial regeneration and tight junction (TJ) reconstruction. Dysregulation of programmed cell death (PCD) pathways, including necroptosis, pyroptosis, ferroptosis, and other cell death modalities, disrupts epithelial integrity and exacerbates inflammatory responses (123). Epithelial cell injury increases barrier permeability, while the mucus layer, which relies on epithelial and goblet cell homeostasis, is also compromised.

Ferroptosis is a form of cell death driven by iron-dependent lipid peroxidation, which induces oxidative stress, severely disrupts intestinal barrier integrity, promotes microbial translocation, and aggravates gut microbial dysbiosis (123, 124). Meanwhile, harmful intestinal bacteria further exacerbate iron overload and lipid peroxidation through metabolite-mediated mechanisms (125).

Natural products, such as tea polyphenols, can inhibit ferroptosis and alleviate colitis symptoms through mechanisms involving the Nrf2/HO-1/GPX4 axis (126). Urolithin A enhances mucus production and improves intestinal barrier function by activating the AhR and Nrf2 pathways (127). EGCG activates the Nrf2-GPX4 pathway, enhances antioxidant defense capacity, and improves iron metabolism through upregulation of ferritin expression, thereby alleviating intestinal inflammation (128). In the present study, the high-frequency co-occurrence of “oxidative stress” (total link strength = 1,726) and “inflammation” (total link strength = 2,368) constituted the core mechanistic background of this field, while ferroptosis-related PCD pathways represent a critical link connecting oxidative stress with barrier disruption. By targeting PCD pathways, natural products contribute to the maintenance of epithelial barrier integrity, representing a transition in this field from classical pathway-based investigations toward cellular regulatory mechanisms.

Pyroptosis represents another critical mechanism. Upon activation by inflammatory stimuli, inflammatory caspases cleave gasdermin D (GSDMD), and the N-terminal fragment of GSDMD inserts into the cell membrane to form pores, triggering cellular swelling, membrane rupture, and the release of intracellular contents, thereby causing structural damage to the intestinal barrier (129). This process promotes the release of inflammatory mediators, exacerbates inflammatory progression (130, 131), disrupts intestinal epithelial barrier function, and delays barrier restoration (132). The natural product isofraxidin reverses pyroptosis-associated alterations by upregulating Nrf2 and reducing oxidative stress levels (133). Ruscogenin decreases the expression of pyroptosis-related proteins and restores epithelial barrier integrity (134, 135). Pelargonidin-3-galactoside effectively alleviates DSS-induced ulcerative colitis by inhibiting pyroptotic responses in intestinal epithelial cells and enhancing the structural integrity of the gut microbiota (136). Although the pyroptosis-related PCD pathway has not yet formed an independent cluster in the keyword landscape of the present study, its mechanistic association with oxidative stress and inflammatory networks has increasingly become a focus of investigation, suggesting that this direction may represent an important emerging research area in the future.

Necroptosis is a form of programmed cell death mediated by the canonical RIPK1–RIPK3–MLKL signaling axis, characterized by plasma membrane rupture, intracellular content release, and strong inflammatory responses (137, 138). Necroptosis in epithelial cells directly disrupts the epithelial monolayer structure, leading to instability of tight junction proteins, including Occludin, Claudin, and ZO-1, and increased intestinal permeability (139, 140). Beyond direct mechanical disruption, the persistent inflammatory environment associated with necroptosis and sustained accumulation of damage-associated molecular patterns (DAMPs) can inhibit epithelial regeneration and repair programs, such as the Wnt/β-catenin and IL-22/STAT3 axes, thereby delaying barrier reconstruction (141). Currently, direct evidence regarding natural product-based inhibitors of necroptosis remains limited, making this an important area requiring further investigation.

PANoptosis has been proposed to describe a coupled form of programmed cell death in which pyroptosis, apoptosis, and necroptosis are simultaneously assembled and activated under strong inflammatory stimulation (142–144). These excessive cell death modalities, driven by molecular crosstalk, collectively contribute to intestinal injury and inflammatory progression. Natural products can simultaneously regulate multiple forms of cell death (144, 145), providing unique therapeutic advantages in this field. Berberine reduces cytokine-induced apoptosis in Caco-2 cells *in vitro* by decreasing JNK phosphorylation and promotes colonic epithelial recovery in DSS-treated mice. In addition, berberine activates the Wnt/β-catenin pathway through regulation of the miR-103a-3p/BRD4 axis, thereby attenuating colitis-induced pyroptosis and intestinal mucosal barrier defects (142, 146). Apple polyphenols extract (APE) simultaneously inhibits pyroptosis and apoptosis, accompanied by parallel improvements in inflammatory status and the recovery of TJ- and mucus layer-related indicators (147). The artemisinin derivative SM934 protects intestinal barrier function by inhibiting epithelial cell apoptosis and pyroptosis through the NLRP3/NF-κB/MAPK signaling axis (148).

Recent bibliometric studies have also highlighted the increasingly important role of research related to the gut microbiota, especially the interactions between environmental factors, microbial changes, and disease progression. Although this study further focuses on the role of natural products in protecting the intestinal barrier and the related mechanisms (149).

The identified research hotspots reflect the increase in scientific attention and research activities, but this does not necessarily mean that a complete consensus has been reached on the mechanisms. Differences in analytical methods and experimental designs may lead to heterogeneity among various studies. Future research that integrates multi-omics methods with standardized technologies will be crucial for further verifying their underlying mechanisms.

## 5 Conclusion

The results show that the research on natural products and intestinal barrier function is in a rapidly developing stage. The research speed has significantly accelerated after 2020, with a total of 1,679 papers included. China leads in terms of productivity and citation impact, but more extensive cross-national and cross-institutional cooperation is still insufficient. Cooperation among countries, institutions and authors should be strengthened to promote the generation of higher-quality research. The current research focus in this field is centered on the intestinal microbiota. Natural products can reshape the microbial community and metabolites, transmitting effects through inflammatory/antioxidant pathways to the chemical and immune barriers, ultimately stabilizing tight junctions and promoting epithelial repair. Among them, the TLR4/TNF-NF-κB-MLCK axis is the key signaling pathway that converts immune inflammation into leakage of tight junctions. Representative compounds such as resveratrol achieve multi-pathway barrier protection through NF-κB-related signal transduction. In summary, this field is shifting from barrier improvement to a comprehensive framework that links the immune network with barrier homeostasis, providing directions for future clinical treatment and new drug development.

## 6 Limitation

Only WoSCC and PubMed was retrieved in this study. Limiting the analysis to only English-language literature may lead to an underestimation of the global impact of academic research conducted in non-English-speaking countries. Countries in East Asia such as China, Japan and South Korea account for a significant portion of global research output in this field. as these findings may not have been adequately considered. Furthermore, bibliometric analysis cannot assess the methodological quality of individual studies, so there is no high level of control over the quality of the included articles. Citation frequency and H-index are widely recognized and commonly used indicators for evaluating academic impact, reflecting the influence and cumulative scientific contributions of publications and researchers. However, these metrics may be affected by factors such as publication age, disciplinary citation patterns, and regional disparities, and therefore cannot fully represent the intrinsic quality or broader impact of research. Moreover, research in this field is published relatively quickly, which means that the current situation captured here may have become outdated by the time it is published, and the timeliness may not be sufficient.

## Data Availability

The original contributions presented in the study are included in the article/**Supplementary Material**. Further inquiries can be directed to the corresponding author.

## References

[1] Almeida A Nayfach S Boland M Strozzi F Beracochea M Shi ZJ . A unified catalog of 204,938 reference genomes from the human gut microbiome. Nat Biotechnol. (2021) 39:105–14. doi: 10.1038/s41587-020-0603-3 32690973 PMC7801254

[2] Neurath MF Artis D Becker C . The intestinal barrier: a pivotal role in health, inflammation, and cancer. Lancet Gastroenterol Hepatol. (2025) 10:573–92. doi: 10.1016/S2468-1253(24)00390-X 40086468

[3] Camilleri M . Leaky gut: mechanisms, measurement and clinical implications in humans. Gut. (2019) 68:1516–26. doi: 10.1136/gutjnl-2019-318427 31076401 PMC6790068

[4] Turner JR . Intestinal mucosal barrier function in health and disease. Nat Rev Immunol. (2009) 9:799–809. doi: 10.1038/nri2653 19855405

[5] Peterson LW Artis D . Intestinal epithelial cells: regulators of barrier function and immune homeostasis. Nat Rev Immunol. (2014) 14:141–53. doi: 10.1038/nri3608 24566914

[6] Pelaseyed T Bergström JH Gustafsson JK Ermund A Birchenough GMH Schütte A . The mucus and mucins of the goblet cells and enterocytes provide the first defense line of the gastrointestinal tract and interact with the immune system. Immunol Rev. (2014) 260:8–20. doi: 10.1111/imr.12182 24942678 PMC4281373

[7] Johansson MEV Phillipson M Petersson J Velcich A Holm L Hansson GC . The inner of the two Muc2 mucin-dependent mucus layers in colon is devoid of bacteria. Proc Natl Acad Sci USA. (2008) 105:15064–9. doi: 10.1073/pnas.0803124105 18806221 PMC2567493

[8] Bevins CL Salzman NH . Paneth cells, antimicrobial peptides and maintenance of intestinal homeostasis. Nat Rev Microbiol. (2011) 9:356–68. doi: 10.1038/nrmicro2546 21423246

[9] Pabst O . New concepts in the generation and functions of IgA. Nat Rev Immunol. (2012) 12:821–32. doi: 10.1038/nri3322 23103985

[10] Iacucci M Santacroce G Majumder S Morael J Zammarchi I Maeda Y . Opening the doors of precision medicine: novel tools to assess intestinal barrier in inflammatory bowel disease and colitis-associated neoplasia. Gut. (2024) 73:1749–62. doi: 10.1136/gutjnl-2023-331579 38851294 PMC11422792

[11] Chelakkot C Ghim J Ryu SH . Mechanisms regulating intestinal barrier integrity and its pathological implications. Exp Mol Med. (2018) 50:1–9. doi: 10.1038/s12276-018-0126-x 30115904 PMC6095905

[12] Sriwastva MK Deng Z-B Wang B Teng Y Kumar A Sundaram K . Exosome-like nanoparticles from Mulberry bark prevent DSS-induced colitis via the AhR/COPS8 pathway. EMBO Rep. (2022) 23:e53365. doi: 10.15252/embr.202153365 34994476 PMC8892346

[13] Zhang M Xu C Liu D Han MK Wang L Merlin D . Oral delivery of nanoparticles loaded with ginger active compound, 6-Shogaol, attenuates ulcerative colitis and promotes wound healing in a murine model of ulcerative colitis. J Crohns Colitis. (2018) 12:217–29. doi: 10.1093/ecco-jcc/jjx115 28961808 PMC5881712

[14] Wang J Ghosh SS Ghosh S . Curcumin improves intestinal barrier function: modulation of intracellular signaling, and organization of tight junctions. Am J Physiol Cell Physiol. (2017) 312:C438–45. doi: 10.1152/ajpcell.00235.2016 28249988 PMC5407015

[15] Fukuda S Toh H Hase K Oshima K Nakanishi Y Yoshimura K . Bifidobacteria can protect from enteropathogenic infection through production of acetate. Nature. (2011) 469:543–7. doi: 10.1038/nature09646 21270894

[16] Rosenthal R Waesch A Masete KV Massarani AS Schulzke J-D Hering NA . The green tea component (-)-epigallocatechin-3-gallate protects against cytokine-induced epithelial barrier damage in intestinal epithelial cells. Front Pharmacol. (2025) 16:1559812. doi: 10.3389/fphar.2025.1559812 40438590 PMC12117334

[17] Ninkov A Frank JR Maggio LA . Bibliometrics: methods for studying academic publishing. Perspect Med Educ. (2022) 11:173–6. doi: 10.1007/s40037-021-00695-4 34914027 PMC9240160

[18] Oh KE Flaherty GT . Travel medicine research in the new millennium: a bibliometric analysis of articles published in Travel Medicine and Infectious Disease, 2003-2019. Travel Med Infect Dis. (2020) 33:101549. doi: 10.1016/j.tmaid.2019.101549 31901399 PMC7128863

[19] Ahmad P Slots J . A bibliometric analysis of periodontology. Periodontol 2000. (2021) 85:237–40. doi: 10.1111/prd.12376 33226679

[20] Albratty M Halawi M Albarrati AM . Natural products for drug discovery in cognitive disabilities: bibliometric hotspots, research trends, conceptual framework, and future directions. Pharm (Basel). (2025) 18:983. doi: 10.3390/ph18070983 40732272 PMC12300366

[21] Mittal G A P Dhali A Prasad R S Y Nurani KM . Plant extracts with antioxidant and hepatoprotective benefits for liver health: a bibliometric analysis of drug delivery systems. World J Gastroenterol. (2025) 31:105836. doi: 10.3748/wjg.v31.i18.105836 40496363 PMC12146923

[22] Liu T Li X Chen Y Li J Wang R Ding Y . Natural products in treating diabetic kidney disease: a visualized bibliometric analysis. Front Pharmacol. (2025) 16:1522074. doi: 10.3389/fphar.2025.1522074 40458808 PMC12127394

[23] Hirsch JE . An index to quantify an individual’s scientific research output. Proc Natl Acad Sci USA. (2005) 102:16569–72. doi: 10.1073/pnas.0507655102 16275915 PMC1283832

[24] Severin A Strinzel M Egger M Barros T Sokolov A Mouatt JV . Relationship between journal impact factor and the thoroughness and helpfulness of peer reviews. PloS Biol. (2023) 21:e3002238. doi: 10.1371/journal.pbio.3002238 37643173 PMC10464996

[25] van Eck NJ Waltman L . Software survey: VOSviewer, a computer program for bibliometric mapping. Scientometrics. (2010) 84:523–38. doi: 10.1007/s11192-009-0146-3 20585380 PMC2883932

[26] van Eck NJ Waltman L van Raan AFJ Klautz RJM Peul WC . Citation analysis may severely underestimate the impact of clinical research as compared to basic research. PloS One. (2013) 8:e62395. doi: 10.1371/journal.pone.0062395 23638064 PMC3634776

[27] Hu Y Wang Y Hong H Chen Y Zhou Q Zhu G . Global trends and prospects related to macrophage in chronic kidney disease: a bibliometric analysis. Ren Fail. (2024) 46:2423846. doi: 10.1080/0886022X.2024.2423846 39572163 PMC11583328

[28] Sapone A Bai JC Ciacci C Dolinsek J Green PH Hadjivassiliou M . Spectrum of gluten-related disorders: consensus on new nomenclature and classification. BMC Med. (2012) 10:13. doi: 10.1186/1741-7015-10-13 22313950 PMC3292448

[29] Roopchand DE Carmody RN Kuhn P Moskal K Rojas-Silva P Turnbaugh PJ . Dietary polyphenols promote growth of the gut bacterium Akkermansia muciniphila and attenuate high-fat diet-induced metabolic syndrome. Diabetes. (2015) 64:2847–58. doi: 10.2337/db14-1916 25845659 PMC4512228

[30] Wu Z Huang S Li T Li N Han D Zhang B . Gut microbiota from green tea polyphenol-dosed mice improves intestinal epithelial homeostasis and ameliorates experimental colitis. Microbiome. (2021) 9:184. doi: 10.1186/s40168-021-01115-9 34493333 PMC8424887

[31] Tomova A Bukovsky I Rembert E Yonas W Alwarith J Barnard ND . The effects of vegetarian and vegan diets on gut microbiota. Front Nutr. (2019) 6:47. doi: 10.3389/fnut.2019.00047 31058160 PMC6478664

[32] Porras D Nistal E Martínez-Flórez S Pisonero-Vaquero S Olcoz JL Jover R . Protective effect of quercetin on high-fat diet-induced non-alcoholic fatty liver disease in mice is mediated by modulating intestinal microbiota imbalance and related gut-liver axis activation. Free Radic Biol Med. (2017) 102:188–202. doi: 10.1016/j.freeradbiomed.2016.11.037 27890642

[33] Bolte LA Vich Vila A Imhann F Collij V Gacesa R Peters V . Long-term dietary patterns are associated with pro-inflammatory and anti-inflammatory features of the gut microbiome. Gut. (2021) 70:1287–98. doi: 10.1136/gutjnl-2020-322670 33811041 PMC8223641

[34] Song D Hao J Fan D . Biological properties and clinical applications of berberine. Front Med. (2020) 14:564–82. doi: 10.1007/s11684-019-0724-6 32335802

[35] Samtiya M Aluko RE Dhewa T Moreno-Rojas JM . Potential health benefits of plant food-derived bioactive components: an overview. Foods. (2021) 10:839. doi: 10.3390/foods10040839 33921351 PMC8068854

[36] Yang Q Liang Q Balakrishnan B Belobrajdic DP Feng Q-J Zhang W . Role of dietary nutrients in the modulation of gut microbiota: a narrative review. Nutrients. (2020) 12:381. doi: 10.3390/nu12020381 32023943 PMC7071260

[37] Gilbert JA Blaser MJ Caporaso JG Jansson JK Lynch SV Knight R . Current understanding of the human microbiome. Nat Med. (2018) 24:392–400. doi: 10.1038/nm.4517 29634682 PMC7043356

[38] Anderson JM Van Itallie CM . Physiology and function of the tight junction. Cold Spring Harb Perspect Biol. (2009) 1:a002584. doi: 10.1101/cshperspect.a002584 20066090 PMC2742087

[39] Gustafsson JK Johansson MEV . The role of goblet cells and mucus in intestinal homeostasis. Nat Rev Gastroenterol Hepatol. (2022) 19:785–803. doi: 10.1038/s41575-022-00675-x 36097076

[40] Yang S Liu H Liu Y . Advances in intestinal epithelium and gut microbiota interaction. Front Microbiol. (2025) 16:1499202. doi: 10.3389/fmicb.2025.1499202 40104591 PMC11914147

[41] Suzuki T . Regulation of the intestinal barrier by nutrients: the role of tight junctions. Anim Sci J. (2020) 91:e13357. doi: 10.1111/asj.13357 32219956 PMC7187240

[42] Hodgkinson K El Abbar F Dobranowski P Manoogian J Butcher J Figeys D . Butyrate’s role in human health and the current progress towards its clinical application to treat gastrointestinal disease. Clin Nutr. (2023) 42:61–75. doi: 10.1016/j.clnu.2022.10.024 36502573

[43] Miao W Wu X Wang K Wang W Wang Y Li Z . Sodium butyrate promotes reassembly of tight junctions in Caco-2 monolayers involving inhibition of MLCK/MLC2 pathway and phosphorylation of PKCβ2. Int J Mol Sci. (2016) 17:1696. doi: 10.3390/ijms17101696 27735862 PMC5085728

[44] Pérez-Reytor D Puebla C Karahanian E García K . Use of short-chain fatty acids for the recovery of the intestinal epithelial barrier affected by bacterial toxins. Front Physiol. (2021) 12:650313. doi: 10.3389/fphys.2021.650313 34108884 PMC8181404

[45] Stan TL Soylu-Kucharz R Burleigh S Prykhodko O Cao L Franke N . Increased intestinal permeability and gut dysbiosis in the R6/2 mouse model of Huntington’s disease. Sci Rep. (2020) 10:18270. doi: 10.1038/s41598-020-75229-9 33106549 PMC7589489

[46] Wang Y Li Y Lin Y Cao C Chen D Huang X . Roles of the gut microbiota in hepatocellular carcinoma: from the gut dysbiosis to the intratumoral microbiota. Cell Death Discov. (2025) 11:140. doi: 10.1038/s41420-025-02413-z 40185720 PMC11971373

[47] Lee T-H Park D Kim YJ Lee I Kim S Oh C-T . Lactobacillus salivarius BP121 prevents cisplatin-induced acute kidney injury by inhibition of uremic toxins such as indoxyl sulfate and p-cresol sulfate via alleviating dysbiosis. Int J Mol Med. (2020) 45:1130–40. doi: 10.3892/ijmm.2020.4495 32124946 PMC7053870

[48] Vivinus-Nébot M Frin-Mathy G Bzioueche H Dainese R Bernard G Anty R . Functional bowel symptoms in quiescent inflammatory bowel diseases: role of epithelial barrier disruption and low-grade inflammation. Gut. (2014) 63:744–52. doi: 10.1136/gutjnl-2012-304066 23878165

[49] Segui-Perez C Stapels DAC Ma Z Su J Passchier E Westendorp B . MUC13 negatively regulates tight junction proteins and intestinal epithelial barrier integrity via protein kinase C. J Cell Sci. (2024) 137:jcs261468. doi: 10.1242/jcs.261468 38345099 PMC10984281

[50] Schroeder BO Birchenough GMH Ståhlman M Arike L Johansson MEV Hansson GC . Bifidobacteria or fiber protects against diet-induced microbiota-mediated colonic mucus deterioration. Cell Host Microbe. (2018) 23:27–40.e7. doi: 10.1016/j.chom.2017.11.004 29276171 PMC5764785

[51] Engevik MA Luk B Chang-Graham AL Hall A Herrmann B Ruan W . Bifidobacterium dentium fortifies the intestinal mucus layer via autophagy and calcium signaling pathways. mBio. (2019) 10:e01087-19. doi: 10.1128/mBio.01087-19 31213556 PMC6581858

[52] Javed NH Alsahly MB Khubchandani J . Oral feeding of probiotic Bifidobacterium infantis: colonic morphological changes in rat model of TNBS-induced colitis. Scientifica (Cairo). (2016) 2016:9572596. doi: 10.1155/2016/9572596 27127686 PMC4834163

[53] Segui-Perez C Huang LZX Paganelli FL Lievens E Strijbis K . Probiotic Bifidobacterium bifidum strains desialylate MUC13 and increase intestinal epithelial barrier function. Sci Rep. (2025) 15:8778. doi: 10.1038/s41598-025-92125-2 40082523 PMC11906825

[54] Odenwald MA Choi W Kuo W-T Singh G Sailer A Wang Y . The scaffolding protein ZO-1 coordinates actomyosin and epithelial apical specializations *in vitro* and *in vivo*. J Biol Chem. (2018) 293:17317–35. doi: 10.1074/jbc.RA118.003908 30242130 PMC6231134

[55] Schwayer C Shamipour S Pranjic-Ferscha K Schauer A Balda M Tada M . Mechanosensation of tight junctions depends on ZO-1 phase separation and flow. Cell. (2019) 179:937–952.e18. doi: 10.1016/j.cell.2019.10.006 31675500

[56] Umeda K Ikenouchi J Katahira-Tayama S Furuse K Sasaki H Nakayama M . ZO-1 and ZO-2 independently determine where claudins are polymerized in tight-junction strand formation. Cell. (2006) 126:741–53. doi: 10.1016/j.cell.2006.06.043 16923393

[57] Odenwald MA Choi W Buckley A Shashikanth N Joseph NE Wang Y . ZO-1 interactions with F-actin and occludin direct epithelial polarization and single lumen specification in 3D culture. J Cell Sci. (2017) 130:243–59. doi: 10.1242/jcs.188185 27802160 PMC5394778

[58] Kuo W-T Zuo L Odenwald MA Madha S Singh G Gurniak CB . The tight junction protein ZO-1 is dispensable for barrier function but critical for effective mucosal repair. Gastroenterology. (2021) 161:1924–39. doi: 10.1053/j.gastro.2021.08.047 34478742 PMC8605999

[59] Shin K Margolis B . ZOning out tight junctions. Cell. (2006) 126:647–9. doi: 10.1016/j.cell.2006.08.005 16923383

[60] da Costa Melo NM de Souza Araújo DF Dantas-Medeiros R da Silva VC de Aragão Tavares E Pinheiro FASD . Flavonoid-rich extract of Cereus jamacaru: safety assessment and protective effects via NF-κB/MAPK modulation in an acute intestinal inflammation model. J Ethnopharmacol. (2026) 369:121818. doi: 10.1016/j.jep.2026.121818 42140532

[61] Li N Shi H Du W Luo S Yang X Yang X . 6-Shogaol ameliorates experimental colitis and colitis-associated colorectal cancer in mice through the Nrf2-Keap1 signaling pathway. Eur J Pharmacol. (2026) 1020:178781. doi: 10.1016/j.ejphar.2026.178781 41825712

[62] Ghosh SS Bie J Wang J Ghosh S . Oral supplementation with non-absorbable antibiotics or curcumin attenuates western diet-induced atherosclerosis and glucose intolerance in LDLR-/- mice--role of intestinal permeability and macrophage activation. PloS One. (2014) 9:e108577. doi: 10.1371/journal.pone.0108577 25251395 PMC4177397

[63] Tian S Guo R Wei S Kong Y Wei X Wang W . Curcumin protects against the intestinal ischemia-reperfusion injury: involvement of the tight junction protein ZO-1 and TNF-α related mechanism. Korean J Physiol Pharmacol. (2016) 20:147–52. doi: 10.4196/kjpp.2016.20.2.147 26937210 PMC4770104

[64] Etienne-Mesmin L Chassaing B Desvaux M De Paepe K Gresse R Sauvaitre T . Experimental models to study intestinal microbes-mucus interactions in health and disease. FEMS Microbiol Rev. (2019) 43:457–89. doi: 10.1093/femsre/fuz013 31162610

[65] Cornick S Tawiah A Chadee K . Roles and regulation of the mucus barrier in the gut. Tissue Barriers. (2015) 3:e982426. doi: 10.4161/21688370.2014.982426 25838985 PMC4372027

[66] Hansson GC . Mucus and mucins in diseases of the intestinal and respiratory tracts. J Intern Med. (2019) 285:479–90. doi: 10.1111/joim.12910 30963635 PMC6497544

[67] Paone P Cani PD . Mucus barrier, mucins and gut microbiota: the expected slimy partners? Gut. (2020) 69:2232–43. doi: 10.1136/gutjnl-2020-322260 32917747 PMC7677487

[68] Hansson GC . Role of mucus layers in gut infection and inflammation. Curr Opin Microbiol. (2012) 15:57–62. doi: 10.1016/j.mib.2011.11.002 22177113 PMC3716454

[69] Wrzosek L Miquel S Noordine M-L Bouet S Joncquel Chevalier-Curt M Robert V . Bacteroides thetaiotaomicron and Faecalibacterium prausnitzii influence the production of mucus glycans and the development of goblet cells in the colonic epithelium of a gnotobiotic model rodent. BMC Biol. (2013) 11:61. doi: 10.1186/1741-7007-11-61 23692866 PMC3673873

[70] Johansson ME Jakobsson HE Holmén-Larsson J Schütte A Ermund A Rodríguez-Piñeiro AM . Normalization of host intestinal mucus layers requires long-term microbial colonization. Cell Host Microbe. (2015) 18:582–92. doi: 10.1016/j.chom.2015.10.007 26526499 PMC4648652

[71] Alves AJT Pereira JA Pansani AHC Magro DO Coy CSR Martinez CAR . Tissue sulfomucin and sialomucin content in colon mucosa without intestinal transit subjected to intervention with Curcuma longa (curcumin). Acta Cir Bras. (2017) 32:182–93. doi: 10.1590/S0102-865020170030000002 28403342

[72] Xun W Shi L Zhou H Hou G Cao T Zhao C . Effects of curcumin on growth performance, jejunal mucosal membrane integrity, morphology and immune status in weaned piglets challenged with enterotoxigenic Escherichia coli. Int Immunopharmacol. (2015) 27:46–52. doi: 10.1016/j.intimp.2015.04.038 25937483

[73] Ghosh SS He H Wang J Gehr TW Ghosh S . Curcumin-mediated regulation of intestinal barrier function: the mechanism underlying its beneficial effects. Tissue Barriers. (2018) 6:e1425085. doi: 10.1080/21688370.2018.1425085 29420166 PMC5823546

[74] Wang Y Li C Li J Zhang S Zhang Q Duan J . Abelmoschus manihot polysaccharide fortifies intestinal mucus barrier to alleviate intestinal inflammation by modulating Akkermansia muciniphila abundance. Acta Pharm Sin B. (2024) 14:3901–15. doi: 10.1016/j.apsb.2024.06.002 39309495 PMC11413673

[75] Mukherjee S Hooper LV . Antimicrobial defense of the intestine. Immunity. (2015) 42:28–39. doi: 10.1016/j.immuni.2014.12.028 25607457

[76] An J Liu Y Wang Y Fan R Hu X Zhang F . The role of intestinal mucosal barrier in autoimmune disease: a potential target. Front Immunol. (2022) 13:871713. doi: 10.3389/fimmu.2022.871713 35844539 PMC9284064

[77] Che Q Luo T Shi J He Y Xu D-L . Mechanisms by which traditional Chinese medicines influence the intestinal flora and intestinal barrier. Front Cell Infect Microbiol. (2022) 12:863779. doi: 10.3389/fcimb.2022.863779 35573786 PMC9097517

[78] Bergstrom K Xia L . The barrier and beyond: roles of intestinal mucus and mucin-type O-glycosylation in resistance and tolerance defense strategies guiding host-microbe symbiosis. Gut Microbes. (2022) 14:2052699. doi: 10.1080/19490976.2022.2052699 35380912 PMC8986245

[79] Ravi A Merlin D Sitaraman SV . Quality is as important as the quantity: role of mucin glycosylation on intestinal barrier function. Gastroenterology. (2007) 133:2065–7. doi: 10.1053/j.gastro.2007.10.049 18054584

[80] Finnie IA Dwarakanath AD Taylor BA Rhodes JM . Colonic mucin synthesis is increased by sodium butyrate. Gut. (1995) 36:93–9. doi: 10.1136/gut.36.1.93 7890244 PMC1382360

[81] Fekete E Buret AG . The role of mucin O-glycans in microbiota dysbiosis, intestinal homeostasis, and host-pathogen interactions. Am J Physiol Gastrointest Liver Physiol. (2023) 324:G452–65. doi: 10.1152/ajpgi.00261.2022 37070751

[82] Takakuwa A Nakamura K Kikuchi M Sugimoto R Ohira S Yokoi Y . Butyric acid and leucine induce α-defensin secretion from small intestinal Paneth cells. Nutrients. (2019) 11:2817. doi: 10.3390/nu11112817 31752111 PMC6893607

[83] Deleu S Machiels K Raes J Verbeke K Vermeire S . Short chain fatty acids and its producing organisms: an overlooked therapy for IBD? EBioMedicine. (2021) 66:103293. doi: 10.1016/j.ebiom.2021.103293 33813134 PMC8047503

[84] Kadry AA El-Antrawy MA El-Ganiny AM . Impact of short chain fatty acids (SCFAs) on antimicrobial activity of new β-lactam/β-lactamase inhibitor combinations and on virulence of Escherichia coli isolates. J Antibiot (Tokyo). (2023) 76:225–35. doi: 10.1038/s41429-023-00595-1 36726014 PMC10040337

[85] Cui X Qiu J Xu Y Guan Z Ding Y Yu S . Mechanisms by which tryptophan metabolites enhance intestinal barrier function to prevent necrotizing enterocolitis in preterm infants. Front Immunol. (2026) 17:1782006. doi: 10.3389/fimmu.2026.1782006 42051527 PMC13111450

[86] Sassone-Corsi M Nuccio S-P Liu H Hernandez D Vu CT Takahashi AA . Microcins mediate competition among Enterobacteriaceae in the inflamed gut. Nature. (2016) 540:280–3. doi: 10.1038/nature20557 27798599 PMC5145735

[87] Schuijt TJ van der Poll T de Vos WM Wiersinga WJ . The intestinal microbiota and host immune interactions in the critically ill. Trends Microbiol. (2013) 21:221–9. doi: 10.1016/j.tim.2013.02.001 23454077

[88] Muzaki ARBM Soncin I Setiagani YA Sheng J Tetlak P Karjalainen K . Long-lived innate IL-17-producing γ/δ T cells modulate antimicrobial epithelial host defense in the colon. J Immunol. (2017) 199:3691–9. doi: 10.4049/jimmunol.1701053 29030488

[89] Cai J Sun L Gonzalez FJ . Gut microbiota-derived bile acids in intestinal immunity, inflammation, and tumorigenesis. Cell Host Microbe. (2022) 30:289–300. doi: 10.1016/j.chom.2022.02.004 35271802 PMC8923532

[90] Lycke NY Bemark M . The regulation of gut mucosal IgA B-cell responses: recent developments. Mucosal Immunol. (2017) 10:1361–74. doi: 10.1038/mi.2017.62 28745325

[91] Xiong N Hu S . Regulation of intestinal IgA responses. Cell Mol Life Sci. (2015) 72:2645–55. doi: 10.1007/s00018-015-1892-4 25837997 PMC4479966

[92] Mantis NJ Forbes SJ . Secretory IgA: arresting microbial pathogens at epithelial borders. Immunol Invest. (2010) 39:383–406. doi: 10.3109/08820131003622635 20450284 PMC3774547

[93] Mantis NJ Rol N Corthésy B . Secretory IgA’s complex roles in immunity and mucosal homeostasis in the gut. Mucosal Immunol. (2011) 4:603–11. doi: 10.1038/mi.2011.41 21975936 PMC3774538

[94] Ghosh S Whitley CS Haribabu B Jala VR . Regulation of intestinal barrier function by microbial metabolites. Cell Mol Gastroenterol Hepatol. (2021) 11:1463–82. doi: 10.1016/j.jcmgh.2021.02.007 33610769 PMC8025057

[95] Kim CH Park J Kim M . Gut microbiota-derived short-chain fatty acids, T cells, and inflammation. Immune Netw. (2014) 14:277–88. doi: 10.4110/in.2014.14.6.277 25550694 PMC4275385

[96] Kim CH . Complex regulatory effects of gut microbial short-chain fatty acids on immune tolerance and autoimmunity. Cell Mol Immunol. (2023) 20:341–50. doi: 10.1038/s41423-023-00987-1 36854801 PMC10066346

[97] Yang W Yu T Huang X Bilotta AJ Xu L Lu Y . Intestinal microbiota-derived short-chain fatty acids regulation of immune cell IL-22 production and gut immunity. Nat Commun. (2020) 11:4457. doi: 10.1038/s41467-020-18262-6 32901017 PMC7478978

[98] Wu W Sun M Chen F Cao AT Liu H Zhao Y . Microbiota metabolite short chain fatty acid acetate promotes intestinal IgA response to microbiota which is mediated by GPR43. Mucosal Immunol. (2017) 10:946–56. doi: 10.1038/mi.2016.114 27966553 PMC5471141

[99] Song G Xie Y Yi L Cheng W Jia H Shi W . Bile acids affect intestinal barrier function through FXR and TGR5. Front Med (Lausanne). (2025) 12:1607899. doi: 10.3389/fmed.2025.1607899 40692955 PMC12277261

[100] Bernardi S Del Bo’ C Marino M Gargari G Cherubini A Andrés-Lacueva C . Polyphenols and intestinal permeability: rationale and future perspectives. J Agric Food Chem. (2020) 68:1816–29. doi: 10.1021/acs.jafc.9b02283 31265272

[101] Li H Gao J Peng W Sun X Qi W Wang Y . Dietary polyphenols-gut microbiota interactions: intervention strategies and metabolic regulation for intestinal diseases. Biology. (2025) 14:1705. doi: 10.3390/biology14121705 41463478 PMC12730813

[102] Khan I Bai Y Zha L Ullah N Ullah H Shah SRH . Mechanism of the gut microbiota colonization resistance and enteric pathogen infection. Front Cell Infect Microbiol. (2021) 11. doi: 10.3389/fcimb.2021.716299 35004340 PMC8733563

[103] Kamada N Kim Y-G Sham HP Vallance BA Puente JL Martens EC . Regulated virulence controls the ability of a pathogen to compete with the gut microbiota. Science. (2012) 336:1325–9. doi: 10.1126/science.1222195 22582016 PMC3439148

[104] Lawley TD Walker AW . Intestinal colonization resistance. Immunology. (2013) 138:1–11. doi: 10.1111/j.1365-2567.2012.03616.x 23240815 PMC3533696

[105] Furne J Springfield J Koenig T DeMaster E Levitt MD . Oxidation of hydrogen sulfide and methanethiol to thiosulfate by rat tissues: a specialized function of the colonic mucosa. Biochem Pharmacol. (2001) 62:255–9. doi: 10.1016/s0006-2952(01)00657-8 11389886

[106] Coddens A Loos M Vanrompay D Remon JP Cox E . Cranberry extract inhibits *in vitro* adhesion of F4 and F18+Escherichia coli to pig intestinal epithelium and reduces *in vivo* excretion of pigs orally challenged with F18+ verotoxigenic E. coli. Vet Microbiol. (2017) 202:64–71. doi: 10.1016/j.vetmic.2017.01.019 28161211

[107] Wan S-Z Liu C Huang C-K Luo F-Y Zhu X . Ursolic acid improves intestinal damage and bacterial dysbiosis in liver fibrosis mice. Front Pharmacol. (2019) 10:1321. doi: 10.3389/fphar.2019.01321 31736766 PMC6838135

[108] Peng L Li Z-R Green RS Holzman IR Lin J . Butyrate enhances the intestinal barrier by facilitating tight junction assembly via activation of AMP-activated protein kinase in Caco-2 cell monolayers. J Nutr. (2009) 139:1619–25. doi: 10.3945/jn.109.104638 19625695 PMC2728689

[109] Chen Y Li K Zhang M Liu J Wang M Xiu Y . Amentoflavone ameliorates DSS-induced ulcerative colitis via gut microbiota-bile acid metabolism axis modulation. J Funct Foods. (2025) 126:106706. doi: 10.1016/j.jff.2025.106706 42574925

[110] Zheng F Sun Y Zhao M Wang R Li W . Research progress on the regulatory effects and mechanisms of natural active products on intestinal barrier function. Front Pharmacol. (2025) 16. doi: 10.3389/fphar.2025.1673568 41480384 PMC12753450

[111] Al-Sadi R Guo S Ye D Rawat M Ma TY . TNF-α modulation of intestinal tight junction permeability is mediated by NIK/IKK-α axis activation of the canonical NF-κB pathway. Am J Pathol. (2016) 186:1151–65. doi: 10.1016/j.ajpath.2015.12.016 26948423 PMC4861759

[112] Shi Z Jiao Y Lai Z Liu J Yang B Hu M . Evaluation of the protective role of resveratrol on LPS-induced septic intestinal barrier function via TLR4/MyD88/NF-κB signaling pathways. Sci Rep. (2025) 15:828. doi: 10.1038/s41598-025-85148-2 39755761 PMC11700184

[113] Pan H-H Zhou X-X Ma Y-Y Pan W-S Zhao F Yu M-S . Resveratrol alleviates intestinal mucosal barrier dysfunction in dextran sulfate sodium-induced colitis mice by enhancing autophagy. World J Gastroenterol. (2020) 26:4945–59. doi: 10.3748/wjg.v26.i33.4945 32952341 PMC7476174

[114] Sahoo DK Heilmann RM Paital B Patel A Yadav VK Wong D . Oxidative stress, hormones, and effects of natural antioxidants on intestinal inflammation in inflammatory bowel disease. Front Endocrinol. (2023) 14. doi: 10.3389/fendo.2023.1217165 37701897 PMC10493311

[115] He F Ru X Wen T . NRF2, a transcription factor for stress response and beyond. Int J Mol Sci. (2020) 21:4777. doi: 10.3390/ijms21134777 32640524 PMC7369905

[116] Peng S Shen L Yu X Zhang L Xu K Xia Y . The role of Nrf2 in the pathogenesis and treatment of ulcerative colitis. Front Immunol. (2023) 14. doi: 10.3389/fimmu.2023.1200111 37359553 PMC10285877

[117] Singh R Chandrashekharappa S Bodduluri SR Baby BV Hegde B Kotla NG . Enhancement of the gut barrier integrity by a microbial metabolite through the Nrf2 pathway. Nat Commun. (2019) 10:89. doi: 10.1038/s41467-018-07859-7 30626868 PMC6327034

[118] Sato T To K Sakurai F Chihara K Warabi E Isobe T . Intestinal epithelial cell-specific restoration of Nrf2 gene in whole-body-knockout mice ameliorates acute colitis. Exp Anim. (2025) 74:335–47. doi: 10.1538/expanim.24-0152 39864840 PMC12270596

[119] Yu X Wang Y Xu Y Li X Zhang J Su Y . Resveratrol attenuates intestinal epithelial barrier dysfunction via Nrf2/HO-1 pathway in dextran sulfate sodium-induced Caco-2 cells. Immun Inflammation Dis. (2024) 12:e1193. doi: 10.1002/iid3.1193 38372468 PMC10875904

[120] Yu X Li X Xu Y Li Y Zhou Y Zhang J . Resveratrol ameliorates ulcerative colitis by upregulating Nrf2/HO-1 pathway activity: integrating animal experiments and network pharmacology. Mol Med Rep. (2024) 29:77. doi: 10.3892/mmr.2024.13201 38488031

[121] Li B Wang Y Jiang X Du H Shi Y Xiu M . Natural products targeting Nrf2/ARE signaling pathway in the treatment of inflammatory bowel disease. Biomedicine Pharmacotherapy. (2023) 164:114950. doi: 10.1016/j.biopha.2023.114950 37263167

[122] Rao R . Oxidative stress-induced disruption of epithelial and endothelial tight junctions. Front Biosci. (2008) 13:7210–26. doi: 10.2741/3223 18508729 PMC6261932

[123] Wu B Su S Li Y Guo L . Dysregulated programmed cell death of intestinal epithelial cells in ulcerative colitis: molecular mechanisms and novel therapeutic interventions (Review). Int J Mol Med. (2025) 56:230. doi: 10.3892/ijmm.2025.5671 41133477 PMC12549076

[124] Xie H Cao C Shu D Liu T Zhang T . The important role of ferroptosis in inflammatory bowel disease. Front Med. (2024) 11. doi: 10.3389/fmed.2024.1449037 39434776 PMC11491328

[125] Zha X Liu X Wei M Huang H Cao J Liu S . Microbiota-derived lysophosphatidylcholine alleviates Alzheimer’s disease pathology via suppressing ferroptosis. Cell Metab. (2025) 37:169–186.e9. doi: 10.1016/j.cmet.2024.10.006 39510074

[126] Li R Li L Wu H Gan H Wu Z Gu R . Tea polyphenols mitigate radiation-induced ferroptosis and intestinal injury by targeting the Nrf2/HO-1/GPX4 signaling pathway. Antioxidants. (2025) 14:580. doi: 10.3390/antiox14050580 40427462 PMC12108355

[127] Yasuda T Takagi T Asaeda K Hashimoto H Kajiwara M Azuma Y . Urolithin A-mediated augmentation of intestinal barrier function through elevated secretory mucin synthesis. Sci Rep. (2024) 14:15706. doi: 10.1038/s41598-024-65791-x 38977770 PMC11231190

[128] Chen J Yin C Zhang Y Lai X Liu C Luo Y . EGCG alleviates DSS-induced colitis by inhibiting ferroptosis through the activation of the Nrf2-GPX4 pathway and enhancing iron metabolism. Nutrients. (2025) 17:547. doi: 10.3390/nu17030547 39940407 PMC11820173

[129] Broz P . Pyroptosis: molecular mechanisms and roles in disease. Cell Res. (2025) 35:334–44. doi: 10.1038/s41422-025-01107-6 40181184 PMC12012027

[130] Wu Y-Z Xie Y Chen L Ning L Hu X-Q Xu X-P . Gasdermin E-mediated intestinal epithelial pyroptosis promotes chemically induced colitis in mice. Gastroenterol Rep (Oxf). (2025) 13:goaf021. doi: 10.1093/gastro/goaf021 40103680 PMC11919448

[131] Bulek K Zhao J Liao Y Rana N Corridoni D Antanaviciute A . Epithelial-derived gasdermin D mediates nonlytic IL-1β release during experimental colitis. J Clin Invest. (2020) 130:4218–34. doi: 10.1172/JCI138103 32597834 PMC7410065

[132] Jena KK Mambu J Boehmer D Sposito B Millet V de Sousa Casal J . Type III interferons induce pyroptosis in gut epithelial cells and impair mucosal repair. Cell. (2024) 187:7533–7550.e23. doi: 10.1016/j.cell.2024.10.010 39500322 PMC11682936

[133] He S Zhang T Wang Y-Y Yuan W Li L Li J . Isofraxidin attenuates dextran sulfate sodium-induced ulcerative colitis through inhibiting pyroptosis by upregulating Nrf2 and reducing reactive oxidative species. Int Immunopharmacol. (2024) 128:111570. doi: 10.1016/j.intimp.2024.111570 38280336

[134] Li J Wu H Zhou J Jiang R Zhuo Z Yang Q . Ruscogenin attenuates ulcerative colitis in mice by inhibiting caspase-1-dependent pyroptosis via the TLR4/NF-κB signaling pathway. Biomedicines. (2024) 12:989. doi: 10.3390/biomedicines12050989 38790951 PMC11117655

[135] Zhao J-W Li L-Y Ye F-W Zhao W-Y . A comprehensive review of food-derived compounds targeting pyroptosis for colitis therapy: from effects to mechanisms. JIR. (2025) 18:11667–88. doi: 10.2147/JIR.S531820 40896541 PMC12399093

[136] Chen J Jiang F Xu N Dong G Jiang J Wang M . Anthocyanin extracted from purple sweet potato alleviates dextran sulfate sodium-induced colitis in mice by suppressing pyroptosis and altering intestinal flora structure. J Med Food. (2024) 27:110–22. doi: 10.1089/jmf.2023.K.0247 38181190

[137] Yao K Shi Z Zhao F Tan C Zhang Y Fan H . RIPK1 in necroptosis and recent progress in related pharmaceutics. Front Immunol. (2025) 16. doi: 10.3389/fimmu.2025.1480027 40007541 PMC11850271

[138] Boonchan M Arimochi H Otsuka K Kobayashi T Uehara H Jaroonwitchawan T . Necroptosis protects against exacerbation of acute pancreatitis. Cell Death Dis. (2021) 12:601. doi: 10.1038/s41419-021-03847-w 34112763 PMC8192754

[139] Negroni A Colantoni E Pierdomenico M Palone F Costanzo M Oliva S . RIP3 AND pMLKL promote necroptosis-induced inflammation and alter membrane permeability in intestinal epithelial cells. Dig Liver Dis. (2017) 49:1201–10. doi: 10.1016/j.dld.2017.08.017 28844856

[140] Liu Y Xu Q Wang Y Liang T Li X Wang D . Necroptosis is active and contributes to intestinal injury in a piglet model with lipopolysaccharide challenge. Cell Death Dis. (2021) 12:62. doi: 10.1038/s41419-020-03365-1 33431831 PMC7801412

[141] Patankar JV Bubeck M Acera MG Becker C . Breaking bad: necroptosis in the pathogenesis of gastrointestinal diseases. Front Immunol. (2023) 14:1203903. doi: 10.3389/fimmu.2023.1203903 37409125 PMC10318896

[142] Zhao C Lin S . PANoptosis in intestinal epithelium: its significance in inflammatory bowel disease and a potential novel therapeutic target for natural products. Front Immunol. (2025) 15. doi: 10.3389/fimmu.2024.1507065 39840043 PMC11747037

[143] Qi Z Zhu L Wang K Wang N . PANoptosis: Emerging mechanisms and disease implications. Life Sci. (2023) 333:122158. doi: 10.1016/j.lfs.2023.122158 37806654

[144] Sun X Yang Y Meng X Li J Liu X Liu H . PANoptosis: Mechanisms, biology, and role in disease. Immunol Rev. (2024) 321:246–62. doi: 10.1111/imr.13279 37823450

[145] Chen B Dong X Zhang JL Sun X Zhou L Zhao K . Natural compounds target programmed cell death (PCD) signaling mechanism to treat ulcerative colitis: a review. Front Pharmacol. (2024) 15:1333657. doi: 10.3389/fphar.2024.1333657 38405669 PMC10885814

[146] Yan F Wang L Shi Y Cao H Liu L Washington MK . Berberine promotes recovery of colitis and inhibits inflammatory responses in colonic macrophages and epithelial cells in DSS-treated mice. Am J Physiol Gastrointest Liver Physiol. (2012) 302:G504–514. doi: 10.1152/ajpgi.00312.2011 22173918 PMC3311435

[147] Liu F Wang X Cui Y Yin Y Qiu D Li S . Apple polyphenols extract (APE) alleviated dextran sulfate sodium induced acute ulcerative colitis and accompanying neuroinflammation via inhibition of apoptosis and pyroptosis. Foods. (2021) 10:2711. doi: 10.3390/foods10112711 34828992 PMC8619666

[148] Shao M Yan Y Zhu F Yang X Qi Q Yang F . Artemisinin analog SM934 alleviates epithelial barrier dysfunction via inhibiting apoptosis and caspase-1-mediated pyroptosis in experimental colitis. Front Pharmacol. (2022) 13. doi: 10.3389/fphar.2022.849014 36120344 PMC9477143

[149] Zhou X-J Chen J-L Hu C-F Li Z-K Zheng H-B Ding T-N . Associations between prevalent unhealthy lifestyles and the gut microbiota: a comprehensive multi-database bibliometric analysis of pathogenic mechanisms and clinical trajectories. Front Med (Lausanne). (2026) 13:1834916. doi: 10.3389/fmed.2026.1834916 42205829 PMC13201409

