# Supplementary material for: The role of natural products in the intestinal barrier: a bibliometric analysis from 2004 to 2025
Source: Front Immunol. 2026 Sep 1;17:1936472. doi: 10.3389/fimmu.2026.1936472 (PMC13574799; doi:10.3389/fimmu.2026.1936472)
Supplement: Supplementary file 1 [file Table1.docx]

Supplementary Material

**Supplementary Table 1.** Search strategy of Web of Science: Science Citation Index Expanded

| **#** | **Searches** |
| --- | --- |
| **1** | TS=(alkaloid OR berberine) |
| **2** | TS=(polyphenol OR theobromine OR quercetin OR resveratrol OR chlorogenic acid OR Epigallocatechin gallate OR EGCG OR baicalin OR baicalein OR wogonoside OR curcumin OR turmeric yellow) |
| **3** | TS=(glycoside OR amygdalin OR almond) |
| **4** | TS=(natural product OR naturally-occurring substance OR natural materials OR natural substances OR natural compound) |
| **5** | #1 OR #2 OR #3 OR #4 |
| **6** | TS=(intestinal barrier OR intestinal barrier function OR intestinal mucosal barrier OR intestinal mucosa barrier OR gut barrier function OR intestinal permeability OR intestinal permeability) |
| **7** | #5 AND #6 |
| **8** | TS=(gastrointestinal absorption OR transport OR permeability OR bioavailability OR nanoparticles OR nanocomposite material OR pharmacokinetics) |
| **9** | #7 NOT #8 |

**Supplementary Table 2.** Search strategy of PubMed

| **#** | **Searches** |
| --- | --- |
| **1** | (alkaloid[Title/Abstract]) OR (berberine[Title/Abstract]) |
| **2** | (polyphenol[Title/Abstract]) OR (theobromine[Title/Abstract]) OR (quercetin[Title/Abstract]) OR (resveratrol[Title/Abstract]) OR (chlorogenic acid[Title/Abstract]) OR (Epigallocatechin gallate[Title/Abstract]) OR (EGCG[Title/Abstract]) OR (baicalin[Title/Abstract]) OR (baicalein[Title/Abstract]) OR (wogonoside[Title/Abstract]) OR (curcumin[Title/Abstract]) OR (turmeric yellow[Title/Abstract]) |
| **3** | (glycoside[Title/Abstract]) OR (amygdalin[Title/Abstract]) OR (almond[Title/Abstract]) |
| **4** | (natural product[Title/Abstract]) OR (naturally-occurring substance[Title/Abstract]) OR (natural materials[Title/Abstract]) OR (natural substances[Title/Abstract]) OR (natural compound[Title/Abstract]) |
| **5** | #1 OR #2 OR #3 OR #4 |
| **6** | (intestinal barrier[Title/Abstract]) OR (intestinal barrier function[Title/Abstract]) OR (intestinal mucosal barrier[Title/Abstract]) OR (intestinal mucosa barrier[Title/Abstract]) OR (gut barrier function[Title/Abstract]) OR (intestinal permeability[Title/Abstract]) OR (intestinal permeability[Title/Abstract]) |
| **7** | #5 AND #6 |
| **8** | (gastrointestinal absorption[Title/Abstract]) OR (transport[Title/Abstract]) OR (permeability[Title/Abstract]) OR (bioavailability[Title/Abstract]) OR (nanoparticles[Title/Abstract]) OR (nanocomposite material[Title/Abstract]) OR (pharmacokinetics[Title/Abstract]) |
| **9** | #7 NOT #8 |
